# Identification of ferroptosis-genes associated with pediatric inflammatory bowel disease bioinformatics and machine learning approaches

**DOI:** 10.3389/fimmu.2025.1619944

**Published:** 2025-11-12

**Authors:** Zhen Xu, Mei Yang, Chenghao Ou, Liming Mao, Zhaoxiu Liu

**Affiliations:** 1 Department of Gastroenterology and Hepatology, Affiliated Hospital of Nantong University, Medical School of Nantong University, Nantong, China; 2 Research Center of Clinical Medicine, Affiliated Hospital of Nantong University, Nantong, China; 3 Department of Immunology, Medical School of Nantong University, Nantong, Jiangsu, China; 4 Basic Medical Research Center, Medical School of Nantong University, Nantong, China; 5 Jiangsu Province Key Laboratory in University for Inflammation and Molecular Drug Target, Nantong, China

**Keywords:** PML, CHAC1, pediatric inflammatory bowel disease, ferroptosis, biomarkers, immune microenvironment, diagnostic model, machine learning

## Abstract

**Background:**

Pediatric inflammatory bowel disease (PIBD) is increasingly common, and early diagnosis remains challenging due to unclear etiology. Ferroptosis, an iron-dependent form of cell death, may be involved in intestinal inflammation, but its expression and role in PIBD are poorly understood.

**Objective:**

To identify ferroptosis-related genes as candidate biomarkers for early diagnosis of PIBD and validate their role in ferroptosis.

**Methods:**

RNA-seq data of PIBD from GEO datasets were analyzed using DESeq2, WGCNA, and functional enrichment analysis. Ferroptosis-related diagnostic genes were screened through LASSO, Random Forest, and mSVM-RFE algorithms, and validated in GSE57945 and GSE117993 datasets. *In vitro* experiments using NCM460 cells were performed to validate the roles of PML and CHAC1 in LPS-induced ferroptosis, including siRNA-mediated gene knockdown, western blotting of ferroptosis-related proteins (ACSL4, SLC7A11, GPX4, FTH), and measurement of lipid peroxidation (MDA levels). CIBERSORT was used to assess immune cell infiltration, and DGIdb was used to predict potential targeted drugs. A ceRNA network was further constructed to explore miRNA-lncRNA interactions regulating these genes.

**Results:**

PML and CHAC1 were identified as potential biomarkers for early diagnosis of PIBD, showing high diagnostic performance (AUC > 0.7) in training, validation, and external datasets. *In vitro* experiments confirmed that knockdown of PML or CHAC1 significantly alleviated LPS-induced ferroptosis in NCM460 cells, as evidenced by restored ferroptosis-related protein expression and reduced MDA accumulation. Consistent with immune infiltration results, both genes were associated with immune-related pathways, and a ceRNA network revealed their potential involvement in complex regulatory mechanisms. DGIdb predicted several candidate drugs targeting these genes.

**Conclusion:**

PML and CHAC1 are promising biomarkers for early PIBD diagnosis. These findings, supported by both bioinformatic analyses and experimental validation, may improve diagnostic accuracy and provide insights into the immune microenvironment and therapeutic strategies.

## 1 Introduction

Inflammatory bowel disease (IBD), including Crohn’s disease (CD) and ulcerative colitis (UC), is a group of chronic inflammatory disorders affecting the intestines. Pediatric IBD (PIBD) accounts for approximately 10% of all IBD cases and is characterized by a more acute disease course and higher risk of severe complications compared with adults (1). Early and accurate diagnosis is critical for effective treatment and improved prognosis, yet remains challenging due to overlapping symptoms with other gastrointestinal disorders and age-dependent variability in disease presentation (2–4).

Ferroptosis, a form of regulated cell death driven by iron-dependent lipid peroxidation, has emerged as a key mechanism in intestinal inflammation and IBD pathogenesis (5–7). However, its role in PIBD remains largely unexplored. Given the immature immune system of pediatric patients, understanding ferroptosis in this context may provide novel insights into disease mechanisms and therapeutic strategies.

**Figure 1:**
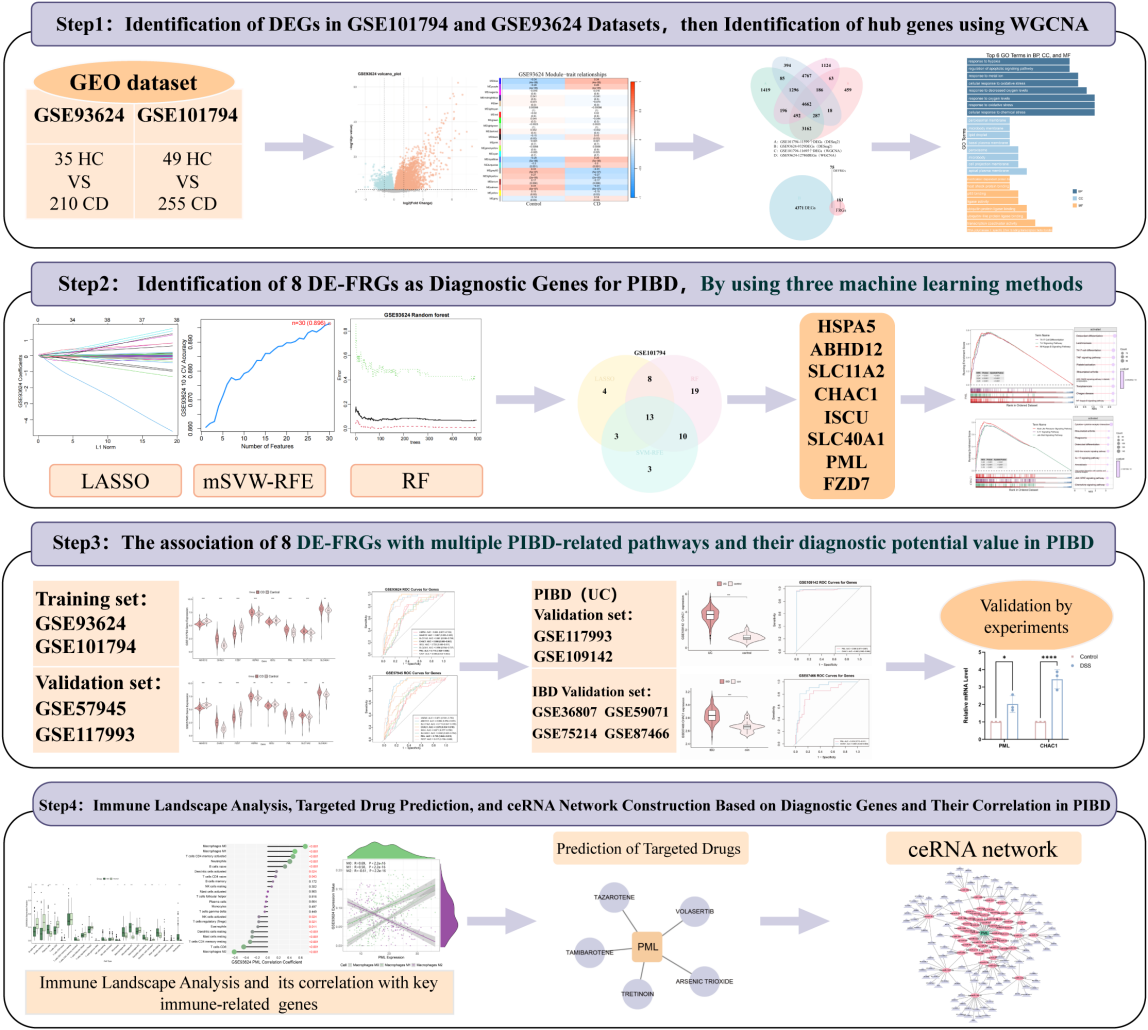
Flowchart. Overview of the workflow for identifying potential PIBD biomarkers, including DEG analysis, WGCNA, machine learning-based selection of key DE-FRGs, pathway analysis, and validation using external datasets.

In this study, we applied machine learning-based screening and external validation to identify ferroptosis-related genes (FRGs) associated with PIBD, highlighting PML and CHAC1 as promising candidates for early diagnostic biomarkers. Our findings aim to provide a theoretical basis for the development of novel molecular tools to improve early detection and clinical management of PIBD (**Figure 1**).

## 2 Materials and methods

### 2.1 Data collection and processing

The RNA sequencing (RNA-seq) data of PIBD were obtained from the GEO database (https://www.ncbi.nlm.nih.gov/geo/) (8). Specifically, GSE101794 contains 304 samples, including 49 healthy controls (HC) and 255 CD samples; while GSE93624 includes 245 samples, with 35 HC samples and 210 CD samples. The validation datasets for key genes include GSE57945 (322 samples, 43 HC and 217 CD samples), which contains 322 samples, and GSE117993 (190 samples, 55 HC and 122 CD samples). Detailed information regarding the datasets is provided in **Supplementary Table 1**. Raw sequencing files in SRA format were processed using a standardized Snakemake-based RNA-seq pipeline. Briefly, FASTQ files were extracted using parallel-fastq-dump, followed by quality control with fastp, alignment to the human genome (GRCh38) using HISAT2, and gene-level quantification using featureCounts. Gene annotation files (GTF) and genome indices were obtained from Ensembl. We used bar plots to display the distribution of sequencing reads across all samples. The results indicate a relatively balanced number of reads among samples, demonstrating consistent sequencing depth and high data quality, which are suitable for subsequent analyses (**Supplementary Figures 1A, B**). Additionally, publicly available FRGs were searched in the FerrDb database (http://www.zhounan.org/ferrdb) to identify genes promoting, inhibiting or marking ferroptosis. A total of 247 FRGs were obtained for subsequent analysis after the removal of duplicates.

### 2.2 Identification of differentially expressed genes and functional enrichment analysis

DEGs between CD and HC groups were identified using the “DESeq2” (9) package in R software. DESeq2 applies the Benjamini-Hochberg (BH) method for multiple testing correction to control the false discovery rate (FDR). DEGs with a corrected p-value (FDR) of < 0.05 and |Log2fold change (FC)| ≥ 0 were considered statistically significant. The “Venn Diagram” package in R software was used to intersect DEGs from the two datasets. Gene Ontology (GO) and Kyoto Encyclopedia of Genes and Genomes (KEGG) enrichment analyses were conducted on common DEGs using the “clusterProfiler” (10) package for both, with a screening criterion of adjusted p-value (P.adj) < 0.05.

### 2.3 Weighted gene co-expression network analysis

WGCNA constructed co-expression modules associated with CD based on gene expression profiles. By selecting an appropriate soft-thresholding power, a scale-free network was established, and a topological overlap matrix (TOM) was calculated for hierarchical clustering to detect gene modules. The module eigengenes (MEs) were then correlated with clinical traits to identify key modules associated with PIBD, from which hub genes were extracted for further analysis. To identify DE-FRGs, the “VennDiagram” package was used to intersect DEGs from the two datasets with genes from the key modules identified in WGCNA.

### 2.4 Identification of potential diagnostic biomarkers

To identify potential diagnostic biomarkers associated with ferroptosis in pediatric inflammatory bowel disease (PIBD), three machine learning algorithms were employed: Least Absolute Shrinkage and Selection Operator (LASSO) regression, Random Forest (RF), and multi–Support Vector Machine Recursive Feature Elimination (mSVM-RFE) (11). LASSO regression was performed with 10-fold cross-validation to select the optimal regularization parameter λ, minimizing the mean cross-validation error and thus preventing overfitting while selecting informative genes. The Random Forest model was constructed using 100 decision trees, and the out-of-bag (OOB) error was used to internally evaluate model performance and stability. The number of trees (ntree) was optimized based on the minimum OOB error. Feature importance was ranked using the Mean Decrease Gini criterion, and the top-ranked genes were selected as candidate biomarkers. mSVM-RFE combined recursive feature elimination with 10-fold cross-validation, iteratively removing less informative features and tuning hyperparameters (cost and gamma) within each fold to identify the optimal feature subset. The expression differences of the selected candidate genes were visualized using violin plots, providing a clear comparison between PIBD patients and healthy controls. Additionally, the diagnostic performance of these genes was evaluated using Receiver Operating Characteristic (ROC) curves, and key metrics including area under the curve (AUC), accuracy, sensitivity, and specificity were calculated. In general, higher AUC values indicate stronger predictive performance of the constructed model.

### 2.5 Single-gene gene set enrichment analysis

To further explore the related pathways of the 8 genes identified single-cell GSEA (12) was performed on target genes using the “gseKEGG” function. First, this study calculated the correlations of the target genes with other genes after the retrieval and extraction of their expression data. The genes were then sorted based on their correlation values (from positive to negative). GSEA analysis was conducted using the sorted gene list, and KEGG pathways were selected for enrichment analysis. Finally, result visualization was conducted via bar charts and lollipop plots to reveal the associated biological pathways.

### 2.6 Immune cell infiltration analysis

“CIBERSORT” (13) was further employed to analyze immune cell infiltration to further investigate PIBD-associated immune responses. “CIBERSORT” is a computational tool used for immune cell composition analysis, which estimates the relative proportions of different immune cell types in a sample based on gene expression data. In our study, this algorithm was utilized to investigate the differences in immune cell infiltration between CD and HC groups. Additionally, Spearman correlation analysis was adopted to explore the association of PML and CHAC1 expression with immune cell infiltration.

### 2.7 Prediction of targeted drugs for diagnostic genes

The DGIdb database was used to further explore potential drugs targeting the screened diagnostic genes, analyzing their interactions with default parameter settings. After that, multiple targeted drugs identified for each diagnostic gene were visualized using Cytoscape software.

### 2.8 Construction of the ceRNA network

To predict mRNA-miRNA interaction pairs based on the eight identified marker genes, miRanda, TargetScan, and miRDB databases were searched. After the identification of results common to all three databases, we searched for the predicted miRNAs in the Spongescan database and filtered for miRNA-lncRNA pairs, to construct a ceRNA network comprising mRNA-miRNA-lncRNA interactions.

### 2.9 qRT-PCR

A total of 20 blood samples were collected in this study, including 10 PIBD patients (aged 13–17 years, with equal numbers of males and females, involvement of colon/ileum, most with active disease and receiving biologic therapy) and 10 healthy controls matched for age and sex, their clinical characteristics are summarized in **Supplementary Table 2**. Total RNA was extracted using the Trizol method, and its concentration was measured before reverse transcription. Next, cDNA was used as a template for qRT-PCR. Finally, the expression data of the target genes was normalized with GAPDH as an internal reference gene. The relative expression of the target genes was determined using the 2^-ΔΔCt^ method. The following primer sequences were used in this experiment:

CHAC1-FORWARD CAGGCACCATGAAGCAGGAGTC CHAC1-REVERSE CTTGAGGGTCGCCGTCGTTTC PML-FORWARD CATCTTCTGCTCCAACCCCAACC PML-REVERSE CTCACTGTGGCTGCTGTCAAGG

### 2.10 Induction of colitis in mice

Three-week-old male C57BL/6 mice were purchased from Shanghai SLAC Laboratory Animal Co., Ltd. (Shanghai, China). A total of ten mice were randomly divided into two groups and acclimatized for one week. Subsequently, five mice were administered 3% (w/v) dextran sulfate sodium (DSS, Macklin, China) in their drinking water continuously for 7 days to induce colitis, while the remaining. Five mice received regular drinking water without DSS and served as controls. Body weight was monitored daily for each mouse. On day 8, all mice were sacrificed for further analyses. All animal experiments were approved by the Ethics Committee of the Affiliated Hospital of Nantong University.

### 2.11 Histopathology and immunohistochemistry

Samples were collected and immediately fixed in 10% neutral-buffered formalin. Paraffin-embedded biopsy sections were prepared for immunohistochemical staining. The primary antibodies used for staining included anti-PML (FNab06574, FineTest) and anti-CHAC1 (FNab11027, FineTest).

### 2.12 Gene knockdown, ferroptosis induction, and related indicator detection

#### 2.12.1 siRNA transfection and knockdown validation

Specific small interfering RNAs (siRNAs) targeting human PML and CHAC1 genes (siPML, siCHAC1) and a non-targeting scrambled negative control siRNA (siNC) were designed and synthesized by GenePharma (Shanghai, China). The human colon epithelial cell line NCM460 was cultured in DMEM medium supplemented with 10% fetal bovine serum (FBS) and 1% penicillin-streptomycin in a 37 °C, 5% CO_2_ incubator. When cells reached 60–70% confluence, transfection was performed using Lipofectamine™ RNAiMAX (Invitrogen, USA) according to the manufacturer’s instructions, with a final siRNA concentration of 50 nM. Knockdown efficiency was assessed by Western blot 48 hours post-transfection. Knockdown experiments for PML and CHAC1 were performed independently, using specific antibodies against PML and CHAC1 (Youpin Biotechnology Company) for detection, with GAPDH (Wuhan Sanying) serving as the loading control.

#### 2.12.2 Ferroptosis induction and indicator detection

To investigate the respective roles of PML and CHAC1 in ferroptosis, we performed independent interventions and detections for each gene. The cell experiment groups were as follows: Control group (transfected with siNC, no LPS treatment); LPS group (transfected with siNC, treated with 1 μg/mL LPS for 24 hours); siPML + LPS group (transfected with siPML, treated with 1 μg/mL LPS for 24 hours); siPML group (transfected with siPML, no LPS treatment). Experiments for CHAC1 used an identical group design (i.e., siCHAC1 + LPS group and siCHAC1 group). To detect ferroptosis-related indicators, total protein was extracted using RIPA lysis buffer containing protease inhibitors. 20–30 μg of protein was separated by SDS-PAGE electrophoresis and transferred to a PVDF membrane. After blocking with 5% skim milk, the membrane was incubated overnight at 4 °C with the following primary antibodies: ACSL4 (Abclonal), SLC7A11 (Wuhan Sanying), FTH (Abcam), GPX4 (Abcam), and GAPDH (Wuhan Sanying). Subsequently, the membrane was incubated with HRP-conjugated secondary antibodies and developed using ECL chemiluminescence. Simultaneously, the lipid peroxidation end product malondialdehyde (MDA) was quantified using the Beyotime (China) TBARS/MDA detection kit. Cell lysates were reacted with thiobarbituric acid, and absorbance was measured at 532 nm. MDA content was normalized to protein concentration and expressed as nmol MDA per mg protein.

## 3 Results

### 3.1 Identification of DEGs in GSE101794 and GSE93624 datasets

A total of 9,329 and 11,599 differentially expressed genes (DEGs) were identified from the GSE93624 and GSE101794 datasets, respectively (**Supplementary Figures 1C, D**). GO and KEGG enrichment analyses revealed that these DEGs are mainly involved in immune regulation, cell signaling, tissue repair, oxidative stress responses, inflammation, and metabolism (**Supplementary Figures 1E, F**).

### 3.2 Identification of hub genes using WGCNA

**Figure 2:**
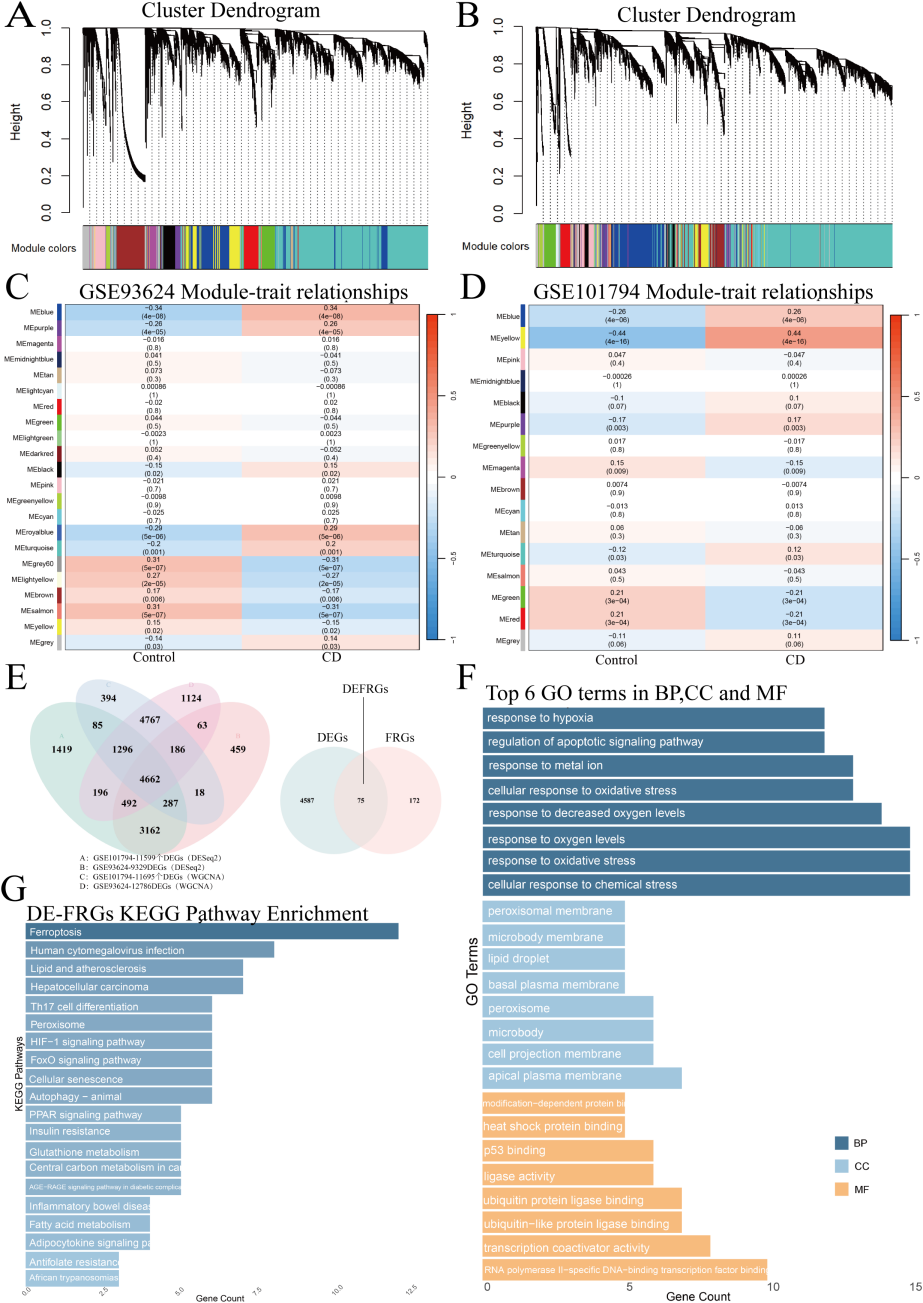
Identification of DEGs and hub genes: **(A–D)** WGCNA analysis identified 16 modules for GSE93624 and 23 modules for GSE101794, with 12,786 and 11,695 genes, respectively. **(E)** Intersection of DEGs from DESeq2 and important module genes from WGCNA resulted in 4,662 genes, which were further intersected with 247 FRGs, yielding 75 DE-FRGs. **(F, G)** GO and KEGG enrichment analyses of the 75 DE-FRGs were performed to explore biological functions and pathways.

WGCNA analysis identified 12,786 genes in GSE93624 and 11,695 genes in GSE101794 (**Figures 2A–D**). After intersecting the DEGs from both datasets with the genes in the significant modules identified by WGCNA, 4,662 common genes were obtained. A total of 75 DE-FRGs were further identified after intersecting these genes with 247 FRGs (**Figure 2E**). GO and KEGG enrichment analyses were conducted to elucidate the biological functions and pathways associated with these DE-FRGs. According to GO enrichments (**Figure 2F**). In terms of biological processes (BP), DE-FRGs were mainly involved in cellular responses to chemical stress, oxygen levels, oxidative stress, and regulation of apoptosis signaling pathways. In molecular functions (MF), DE-FRGs exhibited activities related to transcription regulation, protein modification, and stress response. In cellular components (CC), DE-FRGs were predominantly localized to peroxisomal membranes, microbody membranes, lipid droplets, and cell membranes. Furthermore, as for KEGG pathway enrichment results in **Figure 2G**, DE-FRGs were mainly enriched in pathways related to cell death and survival (e.g., ferroptosis, autophagy, and cellular senescence), metabolic regulation (e.g., glutathione (GSH) metabolism, fatty acid metabolism, and PPAR signaling pathway), and signaling pathways (e.g., HIF-1 signaling pathway, and FoxO signaling pathway). Interestingly, DE-FRGs were also significantly enriched in various immune-related pathways, including Th17 cell differentiation, IBD, and AGE-RAGE signaling pathway, highlighting the broad roles of DE-FRGs in cell metabolism, immune regulation, disease progression, and stress responses.

### 3.3 Identification of 8 DE-FRGs as diagnostic genes for PIBD

**Figure 3:**
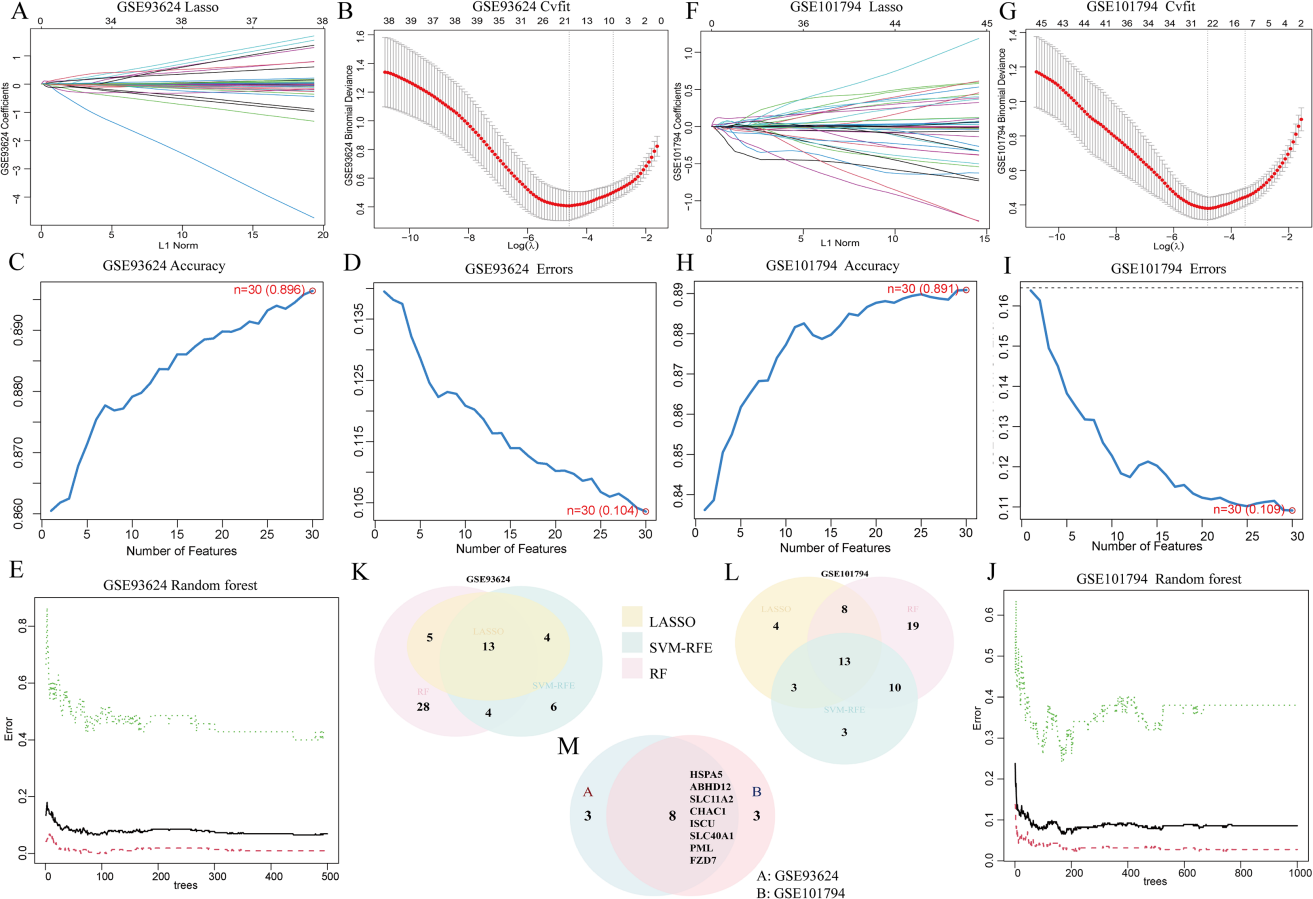
Identification of 8 DE-FRGs as potential diagnostic genes for PIBD using three machine learning methods. **(A–E, L)** LASSO, Random Forest, and mSVM-RFE methods identified 13 candidate genes in the GSE93624 dataset. **(F–J, L)** The same methods identified 13 genes in the GSE101794 dataset. **(M)** The intersection of top-ranked genes from both datasets yielded 8 genes.

Given the differences between PIBD patients and healthy controls, we evaluated the diagnostic potential of DE-FRGs. GSE93624 and GSE101794 were analyzed using LASSO, mSVM-RFE, and RF algorithms to identify key genes distinguishing PIBD from healthy samples. LASSO was performed with 10-fold cross-validation to select the optimal regularization parameter (λ), RF was constructed with 100 decision trees and out-of-bag (OOB) error estimation to ensure model stability, and mSVM-RFE employed 10-fold cross-validation to determine the best-performing feature subset (**Figures 3A–J**).

**Figure 4:**
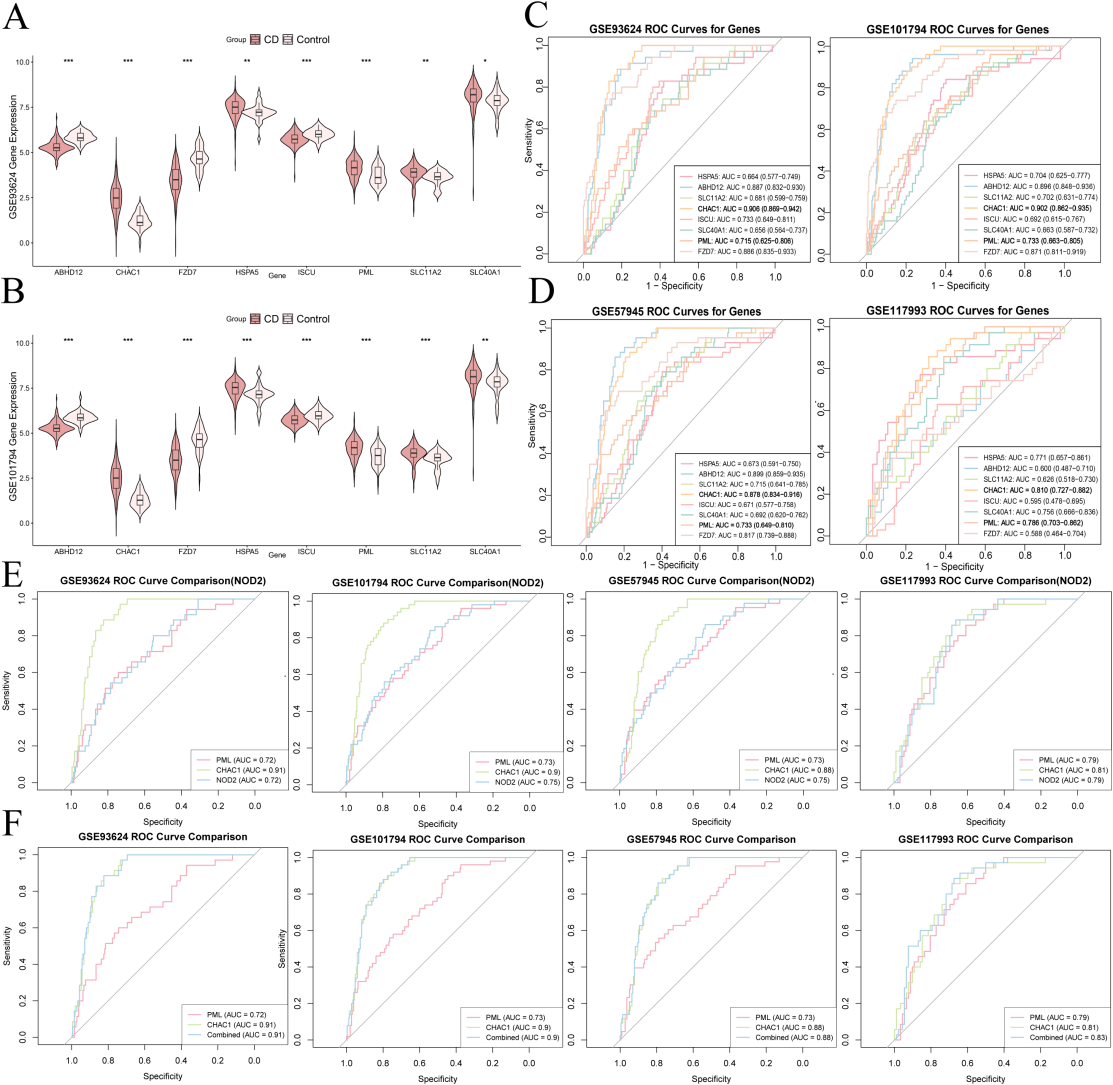
Expression and diagnostic evaluation of key DE-FRGs. **(A, B)** Expression of 8 genes in CD and control groups (***p < 0.001; **p < 0.01; *p < 0.05; ns = not significant). **(C, D)** ROC curves and AUC values for diagnostic performance. **(E)** Comparative AUC of PML, CHAC1, and NOD2. **(F)** Logistic regression models showing superior performance of the PML+CHAC1 combined model.

Cross-analysis of the selected genes from all three models identified HSPA5, ABHD12, SLC11A2, CHAC1, ISCU, SLC40A1, PML, and FZD7 as the main diagnostic candidates for further investigation (**Figures 3K–M**). The expression patterns of these eight genes in PIBD patients and healthy controls were visualized using violin plots (**Figures 4A, B**). In both datasets, all eight genes were significantly different between groups, and consistent results were observed in the validation datasets (**Supplementary Figures 1G, H**). Statistical comparisons were performed using the Wilcoxon test, with significance levels indicated as: *** for p < 0.001, ** for 0.001 ≤ p < 0.01, * for 0.01 ≤ p < 0.05, and ns for p ≥ 0.05.

### 3.4 Diagnostic potential of candidate biomarkers in patients with PIBD

Based on the eight identified diagnostic genes, we assessed their classification performance using the pROC package by calculating ROC curves, AUC values, 95% confidence intervals, and metrics at optimal thresholds (e.g., sensitivity and specificity; **Supplementary Table 3**). All genes exhibited AUC values >0.65 in the training datasets (**Figure 4C**). External validation in GSE57945 and GSE117993 confirmed the diagnostic performance of these genes (**Figure 4D**). Notably, PML and CHAC1 consistently showed strong diagnostic potential in both training and validation datasets, with AUC values exceeding 0.7. Comparison with the established IBD biomarker NOD2 revealed that PML and CHAC1 achieved comparable (14) (15), or even superior, diagnostic accuracy in certain datasets (**Figure 4E**).

To examine whether combining PML and CHAC1 enhances diagnostic accuracy, we constructed a logistic regression model incorporating both genes. Across all four datasets, the combined model consistently achieved AUC values equal to or higher than those of either gene alone (**Figure 4F**). DeLong’s test indicated that the combined model significantly outperformed PML alone in three datasets, while the improvement over CHAC1 alone was not statistically significant. These findings suggest that PML and CHAC1 may function synergistically as diagnostic biomarkers, with statistical support particularly for PML. The relevant information for the DeLong’s test is provided in **Supplementary Table 5**.

**Figure 5:**
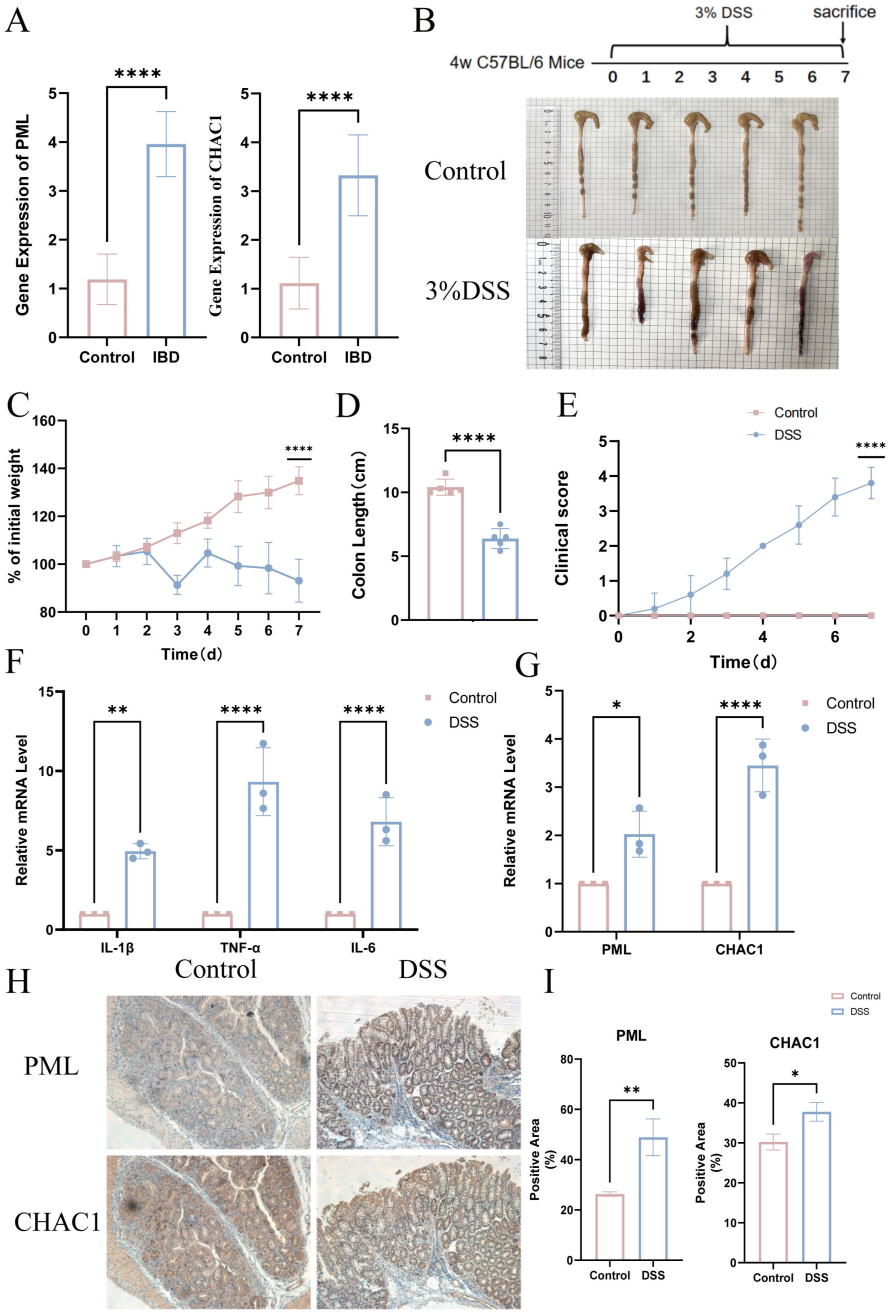
Expression of PML and CHAC1 in human serum and DSS-induced colitis mice. **(A)** Relative mRNA expression in human serum (***p < 0.001). **(B)** Experimental design and representative colonic images of DSS-treated and control mice. **(C–E)** Phenotypic assessment (colon length, body weight, DAI; ***p < 0.001). **(F)** mRNA expression of inflammatory cytokines (***p < 0.001; **p < 0.01; *p < 0.05). **(G)** mRNA expression of PML and CHAC1 in mouse colon tissues. **(H, I)** Immunohistochemical staining and quantification (*p < 0.05; **p < 0.01; ***p < 0.001).

To experimentally validate these bioinformatic findings, we quantified PML and CHAC1 mRNA levels in serum samples from PIBD patients and healthy controls using qRT-PCR. Both genes were significantly upregulated in PIBD patient sera (p < 0.001; **Figure 5A**). In a 4-week DSS-induced colitis mouse model, DSS-treated mice displayed shortened colon length (**Figures 5B, D**; p < 0.0001), weight loss (p < 0.0001; **Figure 5C**), and elevated disease activity index (DAI) scores (p < 0.0001; **Figure 5E**). Colonic tissues of DSS-treated mice showed significantly increased mRNA levels of pro-inflammatory cytokines (IL-6, IL-1β, TNF-α; p < 0.001; **Figure 5F**) as well as PML and CHAC1 (p < 0.01; **Figure 5G**). Immunohistochemistry confirmed elevated protein expression of PML and CHAC1 in the colonic mucosa (**Figures 5H, I**; p < 0.05).

We further evaluated the expression and diagnostic performance of PML and CHAC1 in two independent pediatric IBD datasets (GSE109142 and GSE117993) alongside multiple adult IBD cohorts (**Supplementary Table 4**). Both genes were significantly upregulated in pediatric IBD patients compared to healthy controls (p < 0.001), with ROC analyses yielding AUC values above 0.7. Although these genes were also dysregulated in adult IBD (p < 0.001), their diagnostic performance was more variable, with AUC values ranging from approximately 0.5 to over 0.8 across different adult datasets (**Supplementary Table 4**). We presented the favorable results using violin plots and ROC curves in **Supplementary Figure 5**.

Collectively, these results suggest that while PML and CHAC1 hold diagnostic potential for IBD broadly, their relevance may be particularly heightened in the pediatric context. This can be attributed to the unique vulnerability of the developing intestinal epithelium in children. The maturation of the intestinal mucosal barrier is incomplete, and the antioxidant defense system is relatively underdeveloped, collectively rendering the tissue more susceptible to the very processes—ferroptosis, oxidative stress, and inflammatory injury—in which PML and CHAC1 are functionally implicated. Consequently, the dysregulation of these genes is likely to have a more pronounced pathogenic and diagnostic impact in children, providing a compelling mechanistic rationale for their prioritization as pediatric-specific biomarkers.

### 3.5 Association of diagnostic genes with multiple PIBD-related pathways

**Figure 6:**
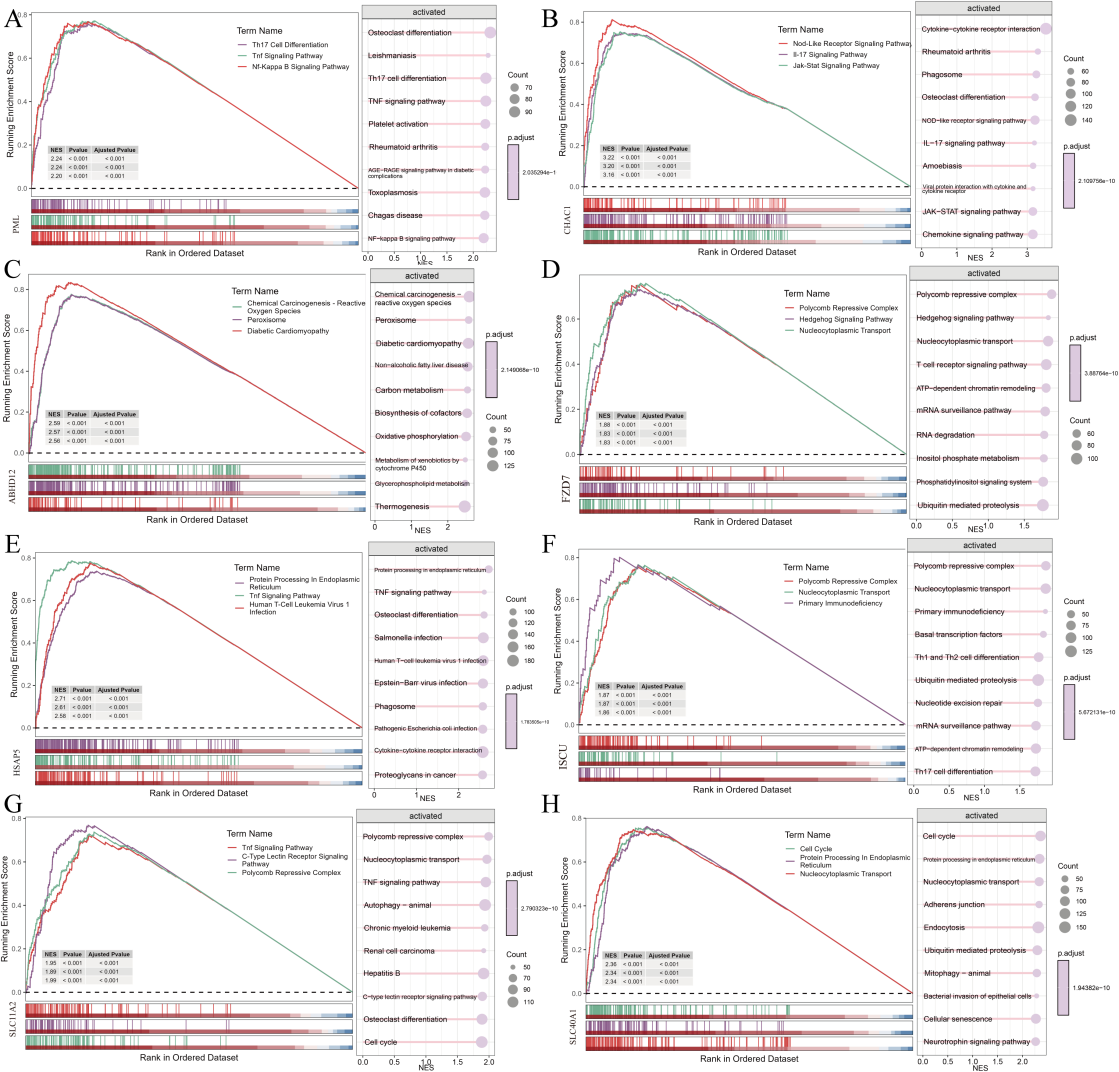
Pathways enriched after ranking the correlated genes of the 8 genes in GSE93624. **(A–H)** Pathways enriched after ranking the correlated genes of the 8 genes in GSE93624.

GSEA-KEGG pathway analysis was conducted to further examine the functional roles of these diagnostic genes in distinguishing PIBD samples from normal samples. As shown in **Figures 6A–H**, the top-10 enriched pathways for each diagnostic gene were involved in various BP, including metabolic regulation and energy balance, immune and inflammatory responses, cancer and cell proliferation, cell death and survival regulation, as well as pathogen interaction. Additionally, the HIF-1 signaling pathway (hypoxia adaptation and cancer metabolism), NF-κB signaling pathway (inflammation and survival), JAK-STAT signaling pathway (immune response and proliferation), and Wnt/FoxO signaling pathway (development and metabolism) integrated multi-domain regulation, linking metabolism, immunity, and disease progression.

### 3.6 Immune landscape analysis and its correlation with key immune-related genes

**Figure 7:**
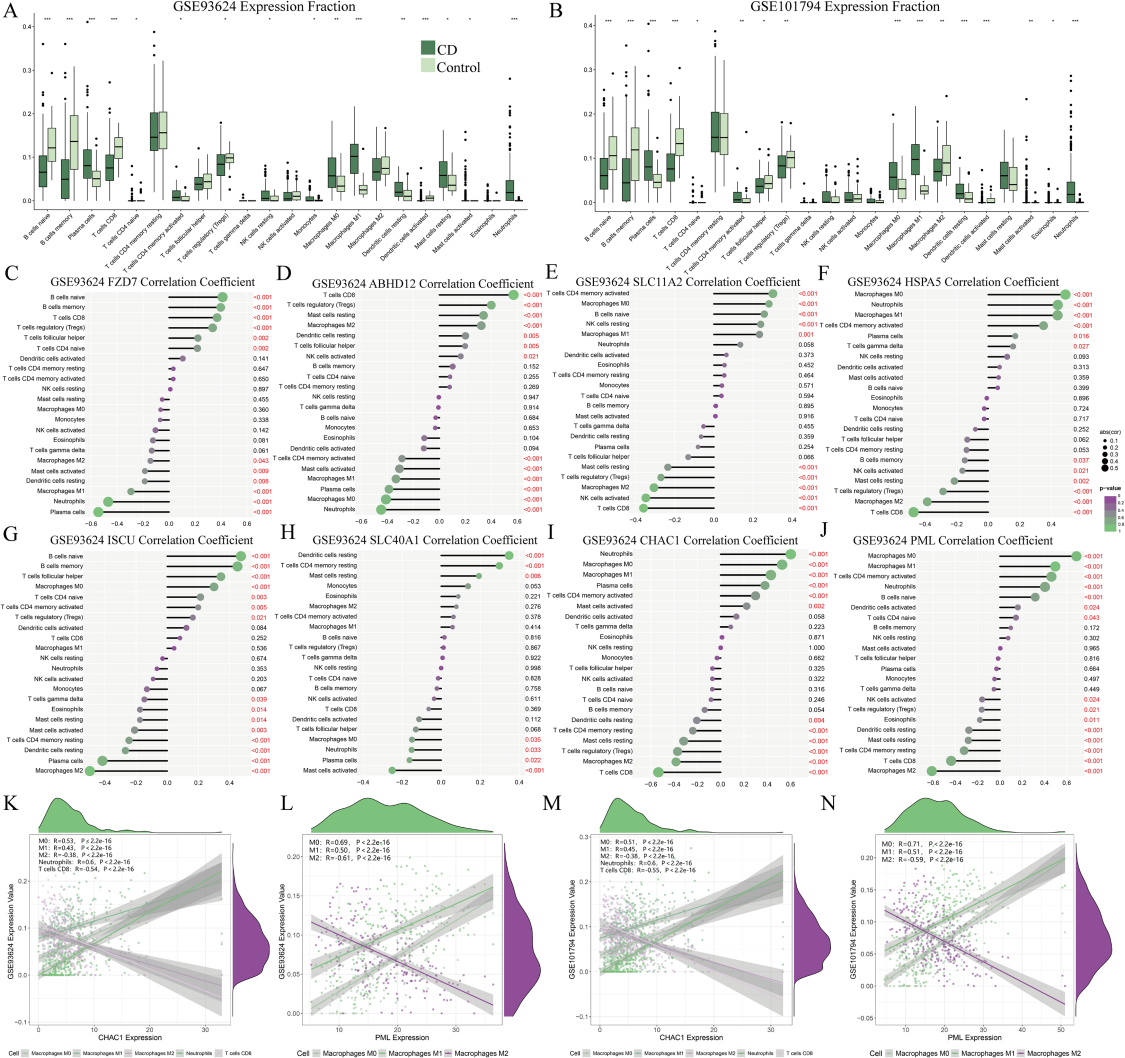
Immune landscape and correlation analysis. **(A, B)** Differences in immune cell infiltration between CD and control groups (***p < 0.001; **p < 0.01; *p < 0.05). **(C–J)** Correlation of immune cell abundance. **(K–N)** Correlation of PML and CHAC1 expression with macrophage subpopulations.

The above results revealed a close relationship between diagnostic genes and immune responses, and a wealth of evidence indicated a strong connection between the immune microenvironment and PIBD. The CIBERSORT algorithm to investigate the differences in the immune microenvironment between PIBD patients and HC. As shown in **Figures 7A, B**, the immune cell landscape of PIBD rectal mucosa differed from that of HC. Compared to the HC group, the CD group exhibited increased infiltration of plasma cells, M0 macrophages, M1 macrophages, activated mast cells, neutrophils, monocytes, activated dendritic cells, and activated CD4+ memory T cells. Conversely, decreased infiltration was observed in M2 macrophages, eosinophils, resting mast cells, CD8+ T cells, naïve CD4+ T cells, naïve B cells, and resting NK cells (p < 0.05). Furthermore, Spearman’s correlation analysis inferred the abundance of infiltrating immune cells (**Figures 7C–J**), with emphasis on PML and CHAC1. The expression of PML was positively correlated with the abundance of M0 macrophages (*r* = 0.69, P = 2.2 × 10^-16^) and M1 macrophages (*r* = 0.50, P = 2.2 × 10^-16^), but negatively correlated with that of M2 macrophages (*r* = -0.61, P = 2.2 × 10^-16^) in the GSE93624 PIBD cohort (p < 0.001). Meanwhile, the expression of CHAC1 was positively correlated with the abundance of M0 and M1 macrophages, and negatively correlated with that of M2 macrophages (p < 0.001). In contrast to PML, CHAC1 expression showed a strong positive correlation with the abundance of neutrophils (*r* = 0.6, P = 2.2 × 10^-16^), but a negative correlation with that of CD8+ T cells (*r* = -0.54, P = 2.2 × 10^-16^). Furthermore, these correlations between PML and CHAC1 with the inferred macrophage Further confirmation in another PIBD cohort (**Figures 7K–N**) revealed overall association of the expression of CHAC1 and PML with pro-inflammatory immune cells (e.g., M0 and M1 macrophages, neutrophils, etc.), suggesting important roles in immune responses and inflammation, with CHAC1 being particularly involved in acute immune challenges. In addition, the negative correlation with M2 macrophages and CD8+ T cells might support their role in modulating the balance of immune responses, potentially exerting an inhibitory effect.

### 3.7 PML and CHAC1 knockdown significantly alleviates LPS-induced ferroptosis

**Figure 8:**
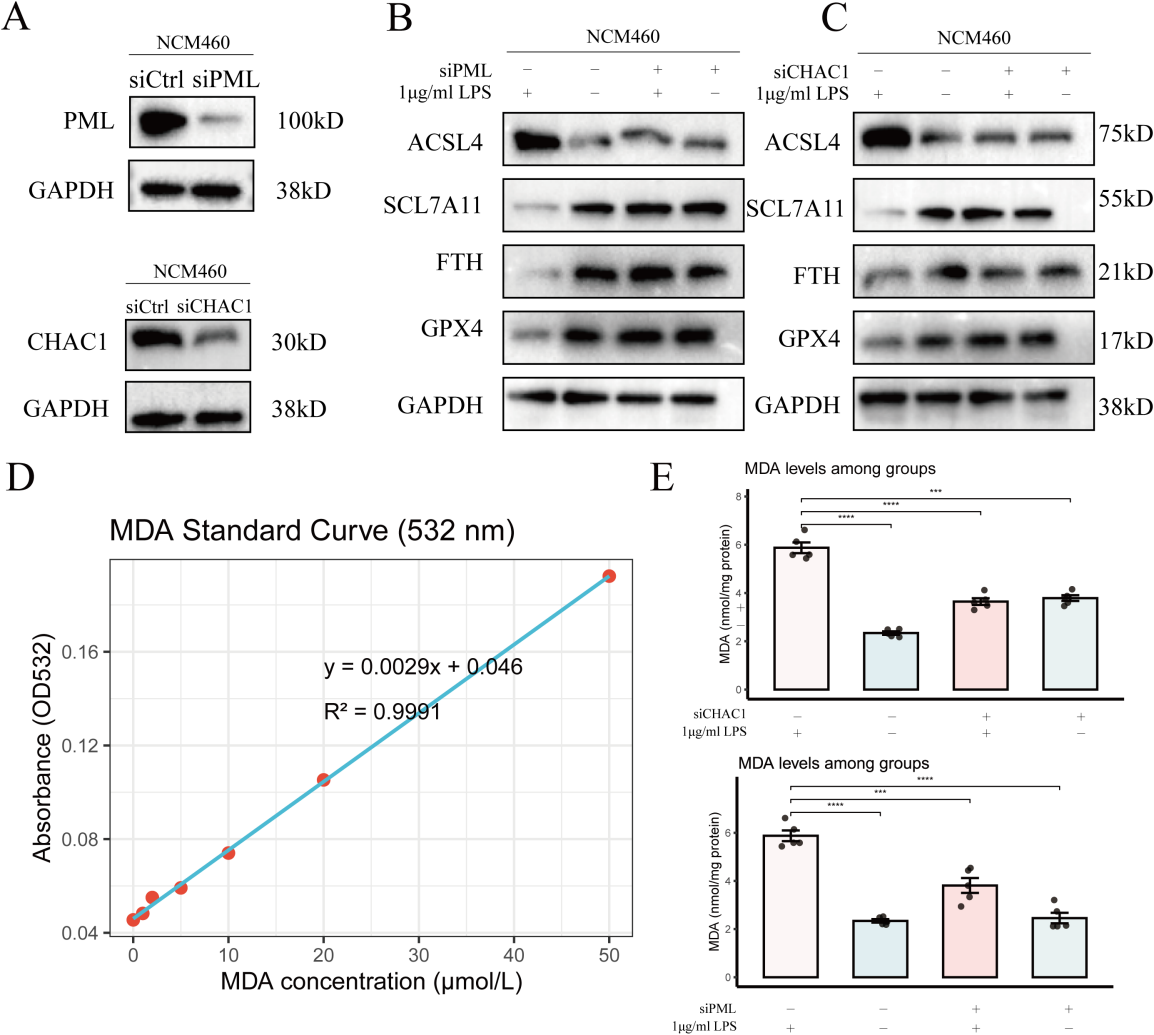
Ferroptosis-related validation of PML and CHAC1. **(A)** Western blot validation of PML and CHAC1 knockdown in NCM460 cells. **(B, C)** Western blot analysis of ferroptosis-related proteins following knockdown of PML and CHAC1 in NCM460 cells. **(D)** Standard curve of malondialdehyde (MDA) measured at 532 nm. **(E)** Quantification of MDA levels after PML and CHAC1 knockdown, indicating increased lipid peroxidation (***P < 0.001, ****P < 0.0001).

To investigate the roles of PML and CHAC1 in inflammation-associated ferroptosis, we performed independent knockdown experiments in NCM460 cells. Western blot analysis confirmed that transfection with specific siRNAs effectively reduced PML and CHAC1 protein levels (**Figure 8A**), indicating successful knockdown.

Subsequently, cells were treated with LPS to induce ferroptosis. As shown in Figure X B, LPS treatment markedly upregulated the ferroptosis-promoting protein ACSL4, downregulated the system Xc− core subunit SLC7A11 and the key antioxidant enzyme GPX4, and depleted the iron storage protein FTH, collectively indicating the occurrence of ferroptosis. Notably, knockdown of PML or CHAC1 prior to LPS stimulation significantly reversed these protein alterations (**Figures 8B, C**).

To further validate the inhibitory effect of gene knockdown on ferroptosis, we measured the levels of the lipid peroxidation end product malondialdehyde (MDA), MDA levels were determined using a standard curve method (**Figure 8D**, MDA standard curve). LPS treatment significantly increased MDA accumulation, whereas knockdown of PML or CHAC1 substantially reduced LPS-induced MDA levels (**Figure 8E**).

Taken together, these results indicate that PML and CHAC1 positively regulate LPS-induced ferroptosis. Their knockdown can reshape the expression profile of ferroptosis-related proteins and effectively suppress lipid peroxidation, thereby enhancing cellular resistance to ferroptotic stress.

### 3.8 Prediction of targeted drugs for diagnostic genes

DGIdb database was visited to further explore potential drugs targeting the diagnostic genes, with their interactions analyzed. **Supplementary Figure 4A** shows the targeted drugs for each diagnostic gene visualized by Cytoscape. This study identified 26 drugs targeting diagnostic genes (**Supplementary Table 6**). Among them, 16 drugs targeted HSPA5, 5 targeted PML, 4 targeted FZD7, and 1 targeted SLC40A1. Unfortunately, no drugs were found to target ABHD12, SLC11A2, CHAC1, or ISCU.

### 3.9 ceRNA network based on diagnostic genes

Based on predictions from the miRanda, TargetScan, miRDB, and SpongeScan databases, we constructed a ceRNA regulatory network centered on eight diagnostic genes. This network includes 445 nodes (8 diagnostic genes, 241 miRNAs, and 196 lncRNAs) and 549 regulatory interactions (**Supplementary Figure 4B**), providing valuable data resources for future mechanistic studies. Detailed information is provided in **Supplementary Table 7**.

Notably, the analysis revealed that miRNAs such as hsa-miR-1207-5p, hsa-miR-1291, and hsa-miR-765 may potentially exert cross-regulatory effects on both CHAC1 and PML (**Supplementary Figures 4B–E**). In addition, lncRNA H19 was predicted to act as a common upstream regulator of both PML and CHAC1, suggesting it may function as a hub in the regulation of these two genes.

Literature reports indicate that lncRNA H19 is significantly upregulated in inflammatory bowel disease and is closely associated with impaired intestinal barrier function, potentially serving as a diagnostic biomarker for IBD (16–18). CHAC1 may participate in the pathogenesis of ulcerative colitis via the miR-214-3p–STAT6 axis (19), while PML may be regulated by miRNAs with immunomodulatory functions, such as miR-146b-3p (20, 21). These literature findings provide supporting evidence for our network predictions.

In summary, the ceRNA network constructed in this study highlights potential regulatory relationships among diagnostic genes, particularly through the coordinated regulation of key nodes such as H19. These predictive results offer new clues and research directions for further exploration of the molecular mechanisms underlying IBD.

## 4 Discussion

### 4.1 PML and CHAC1: potential biomarkers for pibd diagnosis

Pediatric Inflammatory Bowel Disease (PIBD) presents significant diagnostic challenges due to its heterogeneity. Despite advances, many patients still experience poor outcomes, highlighting the need for improved early diagnostic methods. Bioinformatics combined with machine learning offers an effective and cost-efficient approach to identify disease mechanisms and biomarkers. This study investigates the role of ferroptosis in PIBD, identifying key signaling pathways such as Th17 cell differentiation, HIF-1, and FoxO signaling that impact the immune microenvironment. The immune environment in PIBD is dominated by pro-inflammatory responses, with a decrease in anti-inflammatory responses.

PML and CHAC1 were identified as potential diagnostic biomarkers, exhibiting AUC values greater than 0.7 in ROC analysis. They were closely associated with the infiltration of pro-inflammatory immune cells, particularly M0 and M1 macrophages, and neutrophils. These findings suggest that PML and CHAC1 contribute to PIBD pathogenesis by regulating immune responses.

Single-cell RNA sequencing revealed that CHAC1 is primarily expressed in epithelial cells, suggesting its role in epithelial stress and death, while PML is expressed more broadly in epithelial cells, endothelial cells, fibroblasts, and immune cells such as macrophages. This supports PML’s involvement in immune regulation.

Functional studies demonstrated that knockdown of PML and CHAC1 in NCM460 cells alleviated LPS-induced ferroptosis. Knockdown of these genes reversed ferroptosis-related protein changes and reduced lipid peroxidation, indicating they positively regulate ferroptosis and influence cellular resistance to oxidative stress.

In summary, PML and CHAC1 show strong potential as biomarkers for PIBD diagnosis and offer insights into the immune pathogenesis of the disease. Further research into their roles in early diagnosis and treatment response is needed.

### 4.2 PML and CHAC1 are upregulated in PIBD and involved in multiple mechanisms

In recent years, the incidence of PIBD has been steadily increasing, with a pronounced early-onset trend particularly observed in Asian populations. Epidemiological data indicate that PIBD incidence is higher in regions with a high socio-demographic index (SDI), whereas the disease burden is more severe in low SDI areas (22). Regarding pathogenesis, immune dysregulation, genetic abnormalities, and gut microbiota imbalance are widely recognized as key contributing factors. Studies from Chinese researchers utilizing single-cell sequencing and genome-wide association analyses have revealed that cAMP signaling deficiency leads to immune dysfunction, and that PDE4B inhibition by dipyridamole demonstrates therapeutic potential in animal models (23). For diagnosis, fecal calprotectin, a non-invasive biomarker with 3.3. Identification of 8 DE-FRGs as Diagnostic Genes for PIBD high sensitivity, has been extensively employed for monitoring PIBD (24). Treatment strategies are increasingly shifting toward precision medicine, with novel agents including anti-integrins, biologics, and JAK inhibitors gradually entering pediatric clinical trials (25). Furthermore, an elevated long-term risk of malignancy in PIBD patients underscores the importance of rigorous follow-up (26). Future research should focus on elucidating immune regulatory mechanisms and developing individualized therapeutic approaches to improve prognosis and quality of life in affected children.

Ferroptosis is an iron-dependent form of programmed cell death characterized primarily by lipid peroxidation and depletion of glutathione (GSH). In recent years, the pivotal role of ferroptosis in the pathogenesis of IBD has gained increasing attention. Numerous studies have demonstrated that hallmark features of ferroptosis—including iron overload, inactivation of glutathione peroxidase 4 (GPX4), and oxidative stress—are prevalent in tissues from IBD patients as well as in experimental colitis models (27) (28). Administration of ferroptosis inhibitors such as ferrostatin-1 and liproxstatin-1 has been shown to effectively alleviate dextran sulfate sodium (DSS)-induced colitis, ameliorate intestinal inflammation, and preserve mucosal barrier integrity (29). Moreover, complex interactions exist between ferroptosis and the intestinal immune microenvironment. Recent findings indicate that ferroptosis-related gene signatures closely correlate with mucosal immune cell infiltration, including macrophages and CD8^+^ T cells (30). Additionally, gut microbial metabolites, such as short-chain fatty acids and tryptophan derivatives, modulate ferroptotic signaling in intestinal epithelial cells, suggesting that dysbiosis may contribute to mucosal injury via ferroptosis pathways (31). Although direct studies on ferroptosis in PIBD remain limited, the conserved mechanisms elucidated in adult IBD imply that ferroptosis likely plays an important role in PIBD as well.

PML gene functions as a universal sensor of cellular stress, including viral infection, oxidative stress, and DNA damage, capable of initiating diverse protective cellular responses. Mechanistically, PML participates in the regulation and execution of protein complexes through the assembly of PML nuclear bodies, playing critical roles in cell cycle regulation, senescence, and metabolic homeostasis (32). Extensive research has demonstrated that PML exhibits significant tumor suppressor functions across various solid tumors such as breast, lung, and colorectal cancers, with overexpression inducing cell cycle arrest, senescence, and programmed cell death (33–37). In PIBD, the regulatory role of PML is equally crucial. As a key modulator of ferroptosis, alterations in PML expression markedly influence the redox state and survival fate of intestinal epithelial cells, PML overexpression can actively promote ferroptosis by repressing anti-ferroptotic molecules such as SLC7A11 and GPX4 (38). These mechanisms are particularly relevant in PIBD pathology, where the immature immune system and compromised mucosal barrier in children render them more susceptible to epithelial injury and barrier dysfunction triggered by aberrant ferroptosis activation. Furthermore, PML’s involvement in inflammation signaling amplifies its pathological significance in PIBD. Pro-inflammatory cytokines such as TNF-α and IFN-α upregulate PML expression in intestinal tissues of IBD patients, especially in Crohn’s disease (39, 40). PML exacerbates local immune responses and oxidative damage by activating the NLRP3 inflammasome, promoting IL-1β and IL-18 secretion, and increasing ROS production (41). Given PIBD’s heavy reliance on immune response balance, these functions of PML may further drive the vicious cycle of inflammation. Notably, PML also participates in immunometabolic regulation. Its localization at mitochondria-associated membranes (MAMs) influences macrophage polarization and energy metabolism, thereby modulating immune cell functions and the intestinal inflammatory milieu (42). Additionally, PML regulates endothelial cell migration and angiogenesis, processes closely linked to the aberrant vascular remodeling and barrier dysfunction commonly observed in PIBD (42, 43).In summary, PML likely plays a pivotal role in PIBD pathogenesis through multifaceted mechanisms involving ferroptosis regulation, oxidative stress and inflammatory signaling, immunometabolism, and angiogenesis. These immune-metabolic-death network effects are particularly pronounced in pediatric patients. The multilayered regulatory functions of PML not only reveal potential key nodes in PIBD pathophysiology but also provide a theoretical foundation and promising direction for developing PML-targeted biomarkers and precision therapies in early diagnosis and intervention.

CHAC1, a member of the γ-glutamylcyclotransferase family (44), plays a critical role in glutathione (GSH) metabolism and cellular redox homeostasis, primarily by degrading GSH to regulate the intracellular antioxidant system. Studies have shown that CHAC1 overexpression leads to intracellular GSH depletion, significantly inducing oxidative stress and promoting reactive oxygen species (ROS) accumulation, thereby triggering lipid peroxidation and ferroptosis (45–47). By inhibiting the activity of GPX4, CHAC1 further amplifies lipid ROS accumulation, serving as a crucial node in the activation of ferroptotic signaling (48). In adult disease models, CHAC1 has been implicated in the pathogenesis of various chronic pathological conditions, including tumors, inflammation, and organ fibrosis, with its roles in regulating cell death and inflammatory responses gaining increasing attention (49–54). Recent studies demonstrate that silencing CHAC1 in a murine intestinal ischemia-reperfusion injury model significantly inhibits ferroptosis and alleviates oxidative stress-induced tissue damage (55), further suggesting its involvement in modulating intestinal inflammation through ferroptosis regulation.

Although direct research on CHAC1 in PIBD remains limited, mechanistic evidence indicates that CHAC1 may mediate intestinal epithelial dysfunction and mucosal barrier disruption via ferroptosis pathways. In PIBD, where intestinal development is incomplete and antioxidant defenses and iron homeostasis are relatively fragile, ferroptosis is more readily activated, exacerbating inflammatory responses. Given CHAC1’s key role as a rate-limiting factor in GSH metabolism, its expression regulation is likely central to the oxidative stress imbalance and cell death observed in PIBD. Therefore, CHAC1 not only represents a pivotal molecule in ferroptosis regulation but also emerges as a potential novel pathogenic node and therapeutic target in PIBD. Future investigations into CHAC1’s role in intestinal immune homeostasis, barrier integrity, and redox balance will be instrumental in advancing our understanding of PIBD pathogenesis and guiding the development of targeted intervention strategies.

In this study, both PML and CHAC1 were found to be upregulated in PIBD patients. Notably, the combined PML+CHAC1 model demonstrated an improved diagnostic performance compared with PML alone in several datasets, suggesting that PML may serve as a promising indicator associated with PIBD. Although the addition of CHAC1 did not result in a statistically significant improvement, its inclusion appeared to provide complementary information, implying a potential cooperative role between the two genes. Mechanistically, PML and CHAC1 play distinct yet complementary regulatory roles at different levels in ferroptosis and oxidative stress responses. PML primarily acts as an upstream regulator, modulating the ferroptotic microenvironment by controlling reactive oxygen species (ROS) levels, influencing the SLC7A11/GPX4 axis, and activating the NLRP3 inflammasome. Its dual role in promoting inflammation and cell death is particularly pronounced under sustained stimulation of intestinal epithelial cells, where PML activation amplifies oxidative stress and exacerbates inflammatory responses (34, 40, 41). In contrast, CHAC1 functions mainly as a downstream executioner of cellular stress. Induced by ATF4 in the context of endoplasmic reticulum stress and the integrated stress response (ISR), CHAC1’s core function is to degrade glutathione (GSH), thereby impairing the cellular antioxidant defense and directly promoting ferroptosis. CHAC1 activation is considered an irreversible signal for the initiation of programmed cell death. Although PML and CHAC1 act at different initiation points within ferroptosis regulation, they converge on the same pathological endpoint—epithelial cell injury and mucosal barrier disruption.In PIBD, such injury triggers excessive immune responses, leading to gut microbiota dysbiosis and chronic inflammation. This cumulative effect may be exponentially magnified in the susceptible environment of PIBD, resulting in earlier onset and more severe clinical manifestations. This synergistic interaction is especially critical in pediatric patients, whose intestinal barriers are not fully developed and whose immune tolerance mechanisms are relatively weak, making their cells more sensitive to oxidative stress and ferroptosis. Therefore, elevated expression of PML and CHAC1 is not merely a consequence of disease but may represent key drivers of early PIBD pathogenesis. In summary, PML and CHAC1 synergistically activate the ferroptosis pathway from oxidative stress sensing to antioxidant disruption, forming a complementary and amplifying pathogenic network in PIBD development. This mechanistic complementarity provides a theoretical basis for their combined use in early PIBD diagnosis. Future research should further elucidate the regulatory mechanisms of the PML-CHAC1 axis in PIBD and explore joint targeting strategies to offer more precise therapeutic options in clinical practice.

### 4.3 Innovation of this study

This study presents several innovative aspects that distinguish it from previous research. First, unlike most bioinformatics studies that primarily focus on adult IBD, our work specifically investigated pediatric IBD, a population in which molecular-level studies remain scarce despite its rising incidence and clinical importance. Second, we systematically integrated and validated four independent GEO cohorts, thereby enhancing the robustness and reproducibility of our findings. Third, to the best of our knowledge, this is the first study to identify PML and CHAC1 as potential diagnostic biomarkers for PIBD, with consistent diagnostic performance across multiple datasets (AUC > 0.7). Finally, beyond bioinformatics predictions, our findings are further supported by experimental validation in animal and cellular models, including ferroptosis-related experiments, providing translational evidence for their diagnostic relevance. Together, these innovations highlight the novelty of this study and reinforce its clinical significance.

### 4.4 Limitations and future directions

This study identified and validated ferroptosis-related genes (FRGs) in pediatric inflammatory bowel disease (PIBD), but several limitations remain. The relatively small sample size may limit the generalizability of our findings, and the inherent heterogeneity of PIBD could affect the reproducibility of results across different patient populations. Although our bioinformatics analyses were supported by experimental validation, only two genes (PML and CHAC1) were prioritized for experimental confirmation due to their consistently highest diagnostic performance across datasets (AUC > 0.7). Comprehensive functional studies for the remaining candidate genes in larger cohorts and diverse models are still needed to fully assess their biological roles and clinical relevance.

Moreover, the clinical significance of FRGs across different PIBD subtypes has not been fully explored, and their upstream and downstream signaling pathways remain unclear, limiting our understanding of their roles in immune dysregulation and inflammation. Future studies should expand sample sizes, integrate multi-omics data, and utilize advanced experimental models to investigate the mechanisms of PML and CHAC1 in ferroptosis and immune regulation. Validation in larger clinical cohorts and across PIBD subtypes will help confirm their diagnostic and therapeutic potential. Additionally, targeting PML and CHAC1 through gene editing or small-molecule inhibitors may provide new therapeutic strategies, and integrating their expression levels into clinical decision-making could guide personalized treatment approaches to optimize outcomes for PIBD patients.

## 5 Conclusions

PML and CHAC1 are two potential biomarkers for PIBD, both of which are involved in key biological processes such as apoptosis, oxidative stress, and immune regulation that are critical to PIBD pathogenesis. Both genes are closely associated with immune microenvironment changes, particularly in pro-inflammatory immune responses. Furthermore, PML and CHAC1 demonstrate robust diagnostic potential, with high specificity and sensitivity in distinguishing PIBD from healthy samples. Our results indicate that these genes may provide useful insights into the molecular mechanisms underlying PIBD and have potential applications in early diagnosis and disease monitoring. However, further studies are needed to validate their clinical utility and to clarify their functional roles in PIBD progression.

## Data Availability

Publicly available datasets were analyzed in this study. This data can be found here: https://www.ncbi.nlm.nih.gov/geo/GSE93624GSE101794.

## Supplementary material

Supplementary Figure 1 **(A, B)** Distribution of reads in genomic regions (exons, introns, intergenic, mitochondria) for GSE101794 and GSE93624, showing consistent sequencing quality and read distribution. **(C, D)** Volcano plots of DEGs for GSE93624 **(C)** and GSE101794 **(D)**, visualizing gene expression changes vs. statistical significance. **(E, F)** GO and KEGG enrichment analysis of 4,662 DEGs, highlighting pathways related to immune and inflammatory responses, such as immunoglobulin production, leukocyte activation, and viral infections. G-H: Expression levels of 8 genes in CD and control groups from GSE57945 and GSE117993 (***p < 0.001, **p < 0.01, *p < 0.05).

Supplementary Figure 2 **(A–H)** Pathways enriched after ranking the correlated genes of the 8 genes in GSE107794.

Supplementary Figure 3 **(A–H)** The abundance of infiltrating immune cells in GSE101794 using Spearman correlation analysis. The size of the point is proportional to the correlation strength, with the horizontal axis indicating positive or negative correlations, in the figure, groups with p < 0.05 are highlighted in red to indicate statistically significant differences.

Supplementary Figure 4 **(A)** Prediction of targeted drugs for marker genes. **(B)** The ceRNA network, constructed based on the marker genes, comprises 445 nodes, interconnected by 549 edges. **(C, D)** The construction of a ceRNA network based on PML and CHAC1. **(E)** The overlapping nodes in the ceRNA networks of PML and CHAC1.

Supplementary Figure 5 Expression and diagnostic evaluation in pediatric UC and adult IBD. **(A, B)** Gene expression and ROC curves in pediatric UC datasets (***p < 0.001). **(C–F)** Expression and ROC analysis in adult IBD datasets (***p < 0.001).

## References

[1] Bouhuys M Lexmond WS van Rheenen PF . Pediatric inflammatory bowel disease. Pediatrics. (2023) 151:e2022058037. doi: 10.1542/peds.2022-058037, PMID: 36545774

[2] Vuijk SA Camman AE de Ridder L . Considerations in paediatric and adolescent inflammatory bowel disease. J Crohns Colitis. (2024) 18:ii31–45. doi: 10.1093/ecco-jcc/jjae087, PMID: 39475081 PMC11523044

[3] Diez-Martin E Hernandez-Suarez L Muñoz-Villafranca C Martin-Souto L Astigarraga E Ramirez-Garcia A . Inflammatory bowel disease: A comprehensive analysis of molecular bases, predictive biomarkers, diagnostic methods, and therapeutic options. Int J Mol Sci. (2024) 25:7062. doi: 10.3390/ijms25137062, PMID: 39000169 PMC11241012

[4] Bhalla A Shahi A Maity M Safa F Srividya V Clementina R . Inflammatory bowel disease in children: current diagnosis and treatment strategies. Cureus. (2025) 17:e78462. doi: 10.7759/cureus.78462, PMID: 40051947 PMC11883196

[5] Dixon SJ Lemberg KM Lamprecht MR Skouta R Zaitsev EM Gleason CE . Morrison B 3rd, Stockwell BR. Ferroptosis: an iron-dependent form of nonapoptotic cell death. Cell. (2012) 149:1060–72. doi: 10.1016/j.cell.2012.03.042, PMID: 22632970 PMC3367386

[6] Xu S He Y Lin L Chen P Chen M Zhang S . The emerging role of ferroptosis in intestinal disease. Cell Death Dis. (2021) 12:289. doi: 10.1038/s41419-021-03559-1, PMID: 33731703 PMC7969743

[7] Chen Y Zhang P Chen W Chen G . Ferroptosis mediated DSS-induced ulcerative colitis associated with Nrf2/HO-1 signaling pathway. Immunol Lett. (2020) :225:9–15. doi: 10.1016/j.imlet.2020.06.005, PMID: 32540488

[8] Clough E Barrett T . The gene expression omnibus database. Methods Mol Biol. (2016) 1418:93–110. doi: 10.1007/978-1-4939-3578-9_5, PMID: 27008011 PMC4944384

[9] Love MI Huber W Anders S . Moderated estimation of fold change and dispersion for RNA-seq data with DESeq2. Genome Biol. (2014) 15:550. doi: 10.1186/s13059-014-0550-8, PMID: 25516281 PMC4302049

[10] Liberzon A Birger C Thorvaldsdóttir H Ghandi M Mesirov JP Tamayo P . The Molecular Signatures Database (MSigDB) hallmark gene set collection. Cell Syst. (2015) 1:417–25. doi: 10.1016/j.cels.2015.12.004, PMID: 26771021 PMC4707969

[11] Duan KB Rajapakse JC Wang H Azuaje F . Multiple SVM-RFE for gene selection in cancer classification with expression data. IEEE Trans Nanobioscience. (2005) 4:228–34. doi: 10.1109/tnb.2005.853657, PMID: 16220686

[12] Subramanian A Tamayo P Mootha VK Mukherjee S Ebert BL Gillette MA . Gene set enrichment analysis: a knowledge-based approach for interpreting genome-wide expression profiles. Proc Natl Acad Sci U S A. (2005) 102:15545–50. doi: 10.1073/pnas.0506580102, PMID: 16199517 PMC1239896

[13] Newman AM Liu CL Green MR Gentles AJ Feng W Xu Y . Robust enumeration of cell subsets from tissue expression profiles. Nature Methods (2015) 12:453–7. doi: 10.1038/nmeth.3337, PMID: 25822800 PMC4739640

[14] Andreoletti G Shakhnovich V Christenson K Coelho T Haggarty R Afzal NA . Exome analysis of rare and common variants within the NOD signaling pathway. Sci Rep. (2017) 7:46454. doi: 10.1038/srep46454, PMID: 28422189 PMC5396125

[15] Liu Z Zhang Y Jin T Yi C Ocansey DKW Mao F . The role of NOD2 in intestinal immune response and microbiota modulation: A therapeutic target in inflammatory bowel disease. Int Immunopharmacol. (2022) 113:109466. doi: 10.1016/j.intimp.2022.109466, PMID: 36435061

[16] Chen SW Wang PY Liu YC Sun L Zhu J Zuo S . Effect of long noncoding RNA H19 overexpression on intestinal barrier function and its potential role in the pathogenesis of ulcerative colitis. Inflammation Bowel Dis. (2016) 22:2582–92. doi: 10.1097/MIB.0000000000000932, PMID: 27661667

[17] Geng H Bu HF Liu F Wu L Pfeifer K Chou PM . In inflamed intestinal tissues and epithelial cells, interleukin 22 signaling increases expression of H19 long noncoding RNA, which promotes mucosal regeneration. Gastroenterology. (2018) 155:144–55. doi: 10.1053/j.gastro.2018.03.058, PMID: 29621481 PMC6475625

[18] Shaker OG Safa A Khairy A Abozeid NF . Serum long noncoding RNA H19/micro RNA-675-5p axis as a probable diagnostic biomarker in inflammatory bowel disease. Mol Biol Rep. (2023) 50:9029–36. doi: 10.1007/s11033-023-08777-8, PMID: 37716920 PMC10635930

[19] Li JA Wang YD Wang K Wang ZL Jia DY Yang BY . Downregulation of miR-214-3p May Contribute to Pathogenesis of Ulcerative Colitis via Targeting STAT6. BioMed Res Int. (2017) 2017:8524972. doi: 10.1155/2017/8524972, PMID: 28752100 PMC5511677

[20] Nata T Fujiya M Ueno N Moriichi K Konishi H Tanabe H . MicroRNA-146b improves intestinal injury in mouse colitis by activating nuclear factor-κB and improving epithelial barrier function. J Gene Med. (2013) 15:249–60. doi: 10.1002/jgm.2717, PMID: 23813877

[21] Pan Y Wang D Liu F . miR-146b suppresses LPS-induced M1 macrophage polarization via inhibiting the FGL2-activated NF-κB/MAPK signaling pathway in inflammatory bowel disease. Clinics (Sao Paulo). (2022) 77:100069. doi: 10.1016/j.clinsp.2022.100069, PMID: 35749999 PMC9234609

[22] Khan R Kuenzig ME Benchimol EI . Epidemiology of pediatric inflammatory bowel disease. Gastroenterol Clin North Am. (2023) 52:483–96. doi: 10.1016/j.gtc.2023.05.001, PMID: 37543395

[23] Huang B Chen Z Geng L Wang J Liang H Cao Y . Mucosal profiling of pediatric-onset colitis and IBD reveals common pathogenics and therapeutic pathways. Cell. (2019) 179:1160–76. doi: 10.1016/j.cell.2019.10.027, PMID: 31730855

[24] Kapel N Ouni H Benahmed NA Barbot-Trystram L . Fecal calprotectin for the diagnosis and management of inflammatory bowel diseases. Clin Transl Gastroenterol. (2023) 14:e00617. doi: 10.14309/ctg.0000000000000617, PMID: 37440723 PMC10522095

[25] Bretto E Ribaldone DG Caviglia GP Saracco GM Bugianesi E Frara S . Inflammatory bowel disease: emerging therapies and future treatment strategies. Biomedicines. (2023) 11:2249. doi: 10.3390/biomedicines11082249, PMID: 37626745 PMC10452708

[26] Elmahdi R Lemser CE Thomsen SB Allin KH Agrawal M Jess T . Development of cancer among patients with pediatric-onset inflammatory bowel disease: A meta-analysis of population-based studies. JAMA Netw Open. (2022) 5:e220595. doi: 10.1001/jamanetworkopen.2022.0595, PMID: 35230438 PMC8889462

[27] Ocansey DKW Yuan J Wei Z Mao F Zhang Z . Role of ferroptosis in the pathogenesis and as a therapeutic target of inflammatory bowel disease (Review). Int J Mol Med. (2023) 51:53. doi: 10.3892/ijmm.2023.5256, PMID: 37203397 PMC10198063

[28] Xie H Cao C Shu D Liu T Zhang T . The important role of ferroptosis in inflammatory bowel disease. Front Med (Lausanne). (2024) 11:1449037. doi: 10.3389/fmed.2024.1449037, PMID: 39434776 PMC11491328

[29] Zhang X Ma Y Lv G Wang H . Ferroptosis as a therapeutic target for inflammation-related intestinal diseases. Front Pharmacol. (2023) 14:1095366. doi: 10.3389/fphar.2023.1095366, PMID: 36713828 PMC9880170

[30] Tang H Li P Guo X . Ferroptosis-mediated immune microenvironment and therapeutic response in inflammatory bowel disease. DNA Cell Biol. (2023) 42:720–34. doi: 10.1089/dna.2023.0260, PMID: 37943983

[31] Zhou J Lu P He H Zhang R Yang D Liu Q . The metabolites of gut microbiota: their role in ferroptosis in inflammatory bowel disease. Eur J Med Res. (2025) 30:248. doi: 10.1186/s40001-025-02524-4, PMID: 40189555 PMC11974165

[32] Ni Y Chen H Zhan Q Zhuang Z . Nuclear export of PML promotes p53-mediated apoptosis and ferroptosis. Cell Signal. (2024) 121:111278. doi: 10.1016/j.cellsig.2024.111278, PMID: 38944257

[33] Uggè M Simoni M, Fracassi C, Bernardi R M Fracassi C Bernardi R . PML isoforms: a molecular basis for PML pleiotropic functions. Trends Biochem Sci. (2022) 47:609–19. doi: 10.1016/j.tibs.2022.02.002, PMID: 35232626

[34] Guan D Kao HY . The function, regulation and therapeutic implications of the tumor suppressor protein, PML. Cell Biosci. (2015) 5:60. doi: 10.1186/s13578-015-0051-9, PMID: 26539288 PMC4632682

[35] Rego EM Wang ZG Peruzzi D He LZ Cordon-Cardo C Pandolfi PP . Role of promyelocytic leukemia (PML) protein in tumor suppression. J Exp Med. (2001) 193:521–29. doi: 10.1084/jem.193.4.521, PMID: 11181703 PMC2195907

[36] Wu WS Xu ZX Hittelman WN Salomoni P Pandolfi PP Chang KS . Promyelocytic leukemia protein sensitizes tumor necrosis factor alpha-induced apoptosis by inhibiting the NF-kappaB survival pathway. J Biol Chem. (2003) 278:12294–304. doi: 10.1074/jbc.M211849200, PMID: 12540841

[37] Pearson M Carbone R Sebastiani C Cioce M Fagioli M Saito S . PML regulates p53 acetylation and premature senescence induced by oncogenic Ras. Nature. (2000) 406:207–10. doi: 10.1038/35018127, PMID: 10910364

[38] Niwa-Kawakita M Ferhi O Soilihi H Le Bras M Lallemand-Breitenbach V de Thé H . PML is a ROS sensor activating p53 upon oxidative stress. J Exp Med. (2017) 214:3197–206. doi: 10.1084/jem.20160301, PMID: 28931625 PMC5679165

[39] Hsu KS Guan BJ Cheng X Guan D Lam M Hatzoglou M . Translational control of PML contributes to TNFα-induced apoptosis of MCF7 breast cancer cells and decreased angiogenesis in HUVECs. Cell Death Differ. (2016) 23:469–83. doi: 10.1038/cdd.2015.114, PMID: 26383972 PMC5072441

[40] Cheng X Liu Y Chu H Kao HY . Promyelocytic leukemia protein (PML) regulates endothelial cell network formation and migration in response to tumor necrosis factor α (TNFα) and interferon α (IFNα). J Biol Chem. (2012) 287:23356–67. doi: 10.1074/jbc.M112.340505, PMID: 22589541 PMC3390613

[41] Lo YH Huang YW Wu YH Tsai CS Lin YC Mo ST . Selective inhibition of the NLRP3 inflammasome by targeting to promyelocytic leukemia protein in mouse and human. Blood. (2013) 121:3185–94. doi: 10.1182/blood-2012-05-432104, PMID: 23430110

[42] Maarifi G Chelbi-Alix MK Nisole S . PML control of cytokine signaling. Cytokine Growth Factor Rev. (2014) 25:551–61. doi: 10.1016/j.cytogfr.2014.04.008, PMID: 24861946

[43] Eder P Korybalska K Linke K Witowski J . Angiogenesis-related proteins–their role in the pathogenesis and treatment of inflammatory bowel disease. Curr Protein Pept Sci. (2015) 16:249–58. doi: 10.2174/1389203716666150224150756, PMID: 25707471

[44] Kaur A Gautam R Srivastava R Chandel A Kumar A Karthikeyan S . ChaC2, an enzyme for slow turnover of cytosolic glutathione. J Biol Chem. (2017) 292:638–51. doi: 10.1074/jbc.M116.727479, PMID: 27913623 PMC5241738

[45] Mungrue IN Pagnon J Kohannim O Gargalovic PS Lusis AJ . CHAC1/MGC4504 is a novel proapoptotic component of the unfolded protein response, downstream of the ATF4-ATF3-CHOP cascade. J Immunol. (2009) 182:466–76. doi: 10.4049/jimmunol.182.1.466, PMID: 19109178 PMC2846782

[46] Pathria G Ma CT Olson SH Scott D Murad R Ruppin E . NRF2 mediates melanoma addiction to GCDH by modulating apoptotic signalling. Nat Cell Biol. (2022) 24:1422–32. doi: 10.1038/s41556-022-00985-x, PMID: 36050469 PMC9977532

[47] Zhang X Li W Ma Y Zhao X He L Sun P . High-fat diet aggravates colitis-associated carcinogenesis by evading ferroptosis in the ER stress-mediated pathway. Free Radic Biol Med. (2021) 177:156–66. doi: 10.1016/j.freeradbiomed.2021.10.022, PMID: 34688836

[48] Sun J Ren H Wang J Xiao X Zhu L Wang Y . CHAC1: a master regulator of oxidative stress and ferroptosis in human diseases and cancers. Front Cell Dev Biol. (2024) 12:1458716. doi: 10.3389/fcell.2024.1458716, PMID: 39534397 PMC11554486

[49] Li D Liu S Xu J Chen L Xu C Chen F . Ferroptosis-related gene CHAC1 is a valid indicator for the poor prognosis of kidney renal clear cell carcinoma. J Cell Mol Med. (2021) 25:3610–21. doi: 10.1111/jcmm.16458, PMID: 33728749 PMC8034464

[50] Wada Y Takemura K Tummala P Uchida K Kitagaki K Furukawa A . *Helicobacter pylori* induces somatic mutations in *TP53* via overexpression of CHAC1 in infected gastric epithelial cells. FEBS Open Bio. (2018) 8:671–9. doi: 10.1002/2211-5463.12402, PMID: 29632819 PMC5881537

[51] Wanbiao Z Jing M Shi Z Tengxiang C Xueke Z Haiyang L . MIA3 promotes the degradation of GSH (glutathione) by binding to CHAC1, thereby promoting the progression of hepatocellular carcinoma. Mol Cell Biochem. (2024) 479:2769–84. doi: 10.1007/s11010-023-04850-9, PMID: 37948019 PMC11455670

[52] Allawzi A McDermott I Delaney C Nguyen K Banimostafa L Trumpie A . Redistribution of EC-SOD resolves bleomycin-induced inflammation *via* increased apoptosis of recruited alveolar macrophages. FASEB J. (2019) 33:13465–75. doi: 10.1096/fj.201901038RR, PMID: 31560857 PMC6894081

[53] Perra L Balloy V Foussignière T Moissenet D Petat H Mungrue IN . CHAC1 is differentially expressed in normal and cystic fibrosis bronchial epithelial cells and regulates the inflammatory response induced by pseudomonas aeruginosa. Front Immunol. (2018) 9:2823. doi: 10.3389/fimmu.2018.02823, PMID: 30555487 PMC6282009

[54] Yao H Wang Y Zhou W Xu Ce Ge X Zhu J . Chac1 silencing mitigates hemorrhagic shock-induced intestinal injury by inhibiting oxidative stress and ferroptosis. Signa Vitae. (2023) 19:184–93. doi: 10.22514/sv.2023.113

[55] Ricciuto A Mack DR Huynh HQ Jacobson K Otley AR deBruyn J . Diagnostic delay is associated with complicated disease and growth impairment in paediatric crohn’s disease. J Crohns Colitis. (2021) 15:419–31. doi: 10.1093/ecco-jcc/jjaa197, PMID: 32978629 PMC7944510

