# Supplementary figures and images for: Identification of ferroptosis-genes associated with pediatric inflammatory bowel disease bioinformatics and machine learning approaches
Source: Front Immunol. 2025 Nov 12;16:1619944. doi: 10.3389/fimmu.2025.1619944 (PMC12647037; doi:10.3389/fimmu.2025.1619944)

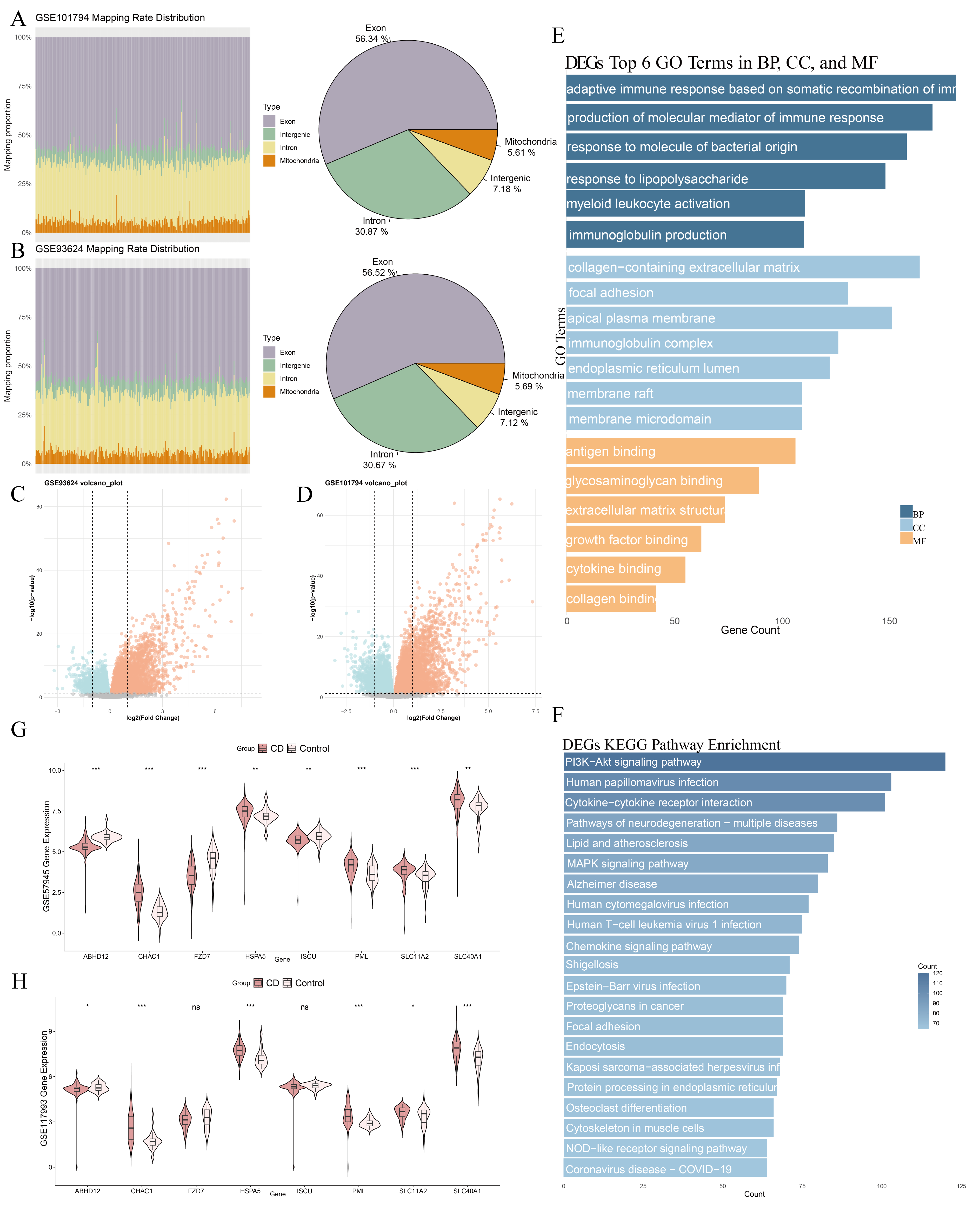

Supplement: Supplementary Figure 1 — (A, B) Distribution of reads in genomic regions (exons, introns, intergenic, mitochondria) for GSE101794 and GSE93624, showing consistent sequencing quality and read distribution. (C, D) Volcano plots of DEGs for GSE93624 (C) and GSE101794 (D), visualizing gene expression changes vs. statistical significance. (E, F) GO and KEGG enrichment analysis of 4,662 DEGs, highlighting pathways related to immune and inflammatory responses, such as immunoglobulin production, leukocyte activation, and viral infections. G-H: Expression levels of 8 genes in CD and control groups from GSE57945 and GSE117993 (***p < 0.001, **p < 0.01, *p < 0.05). [file Image1.tif]

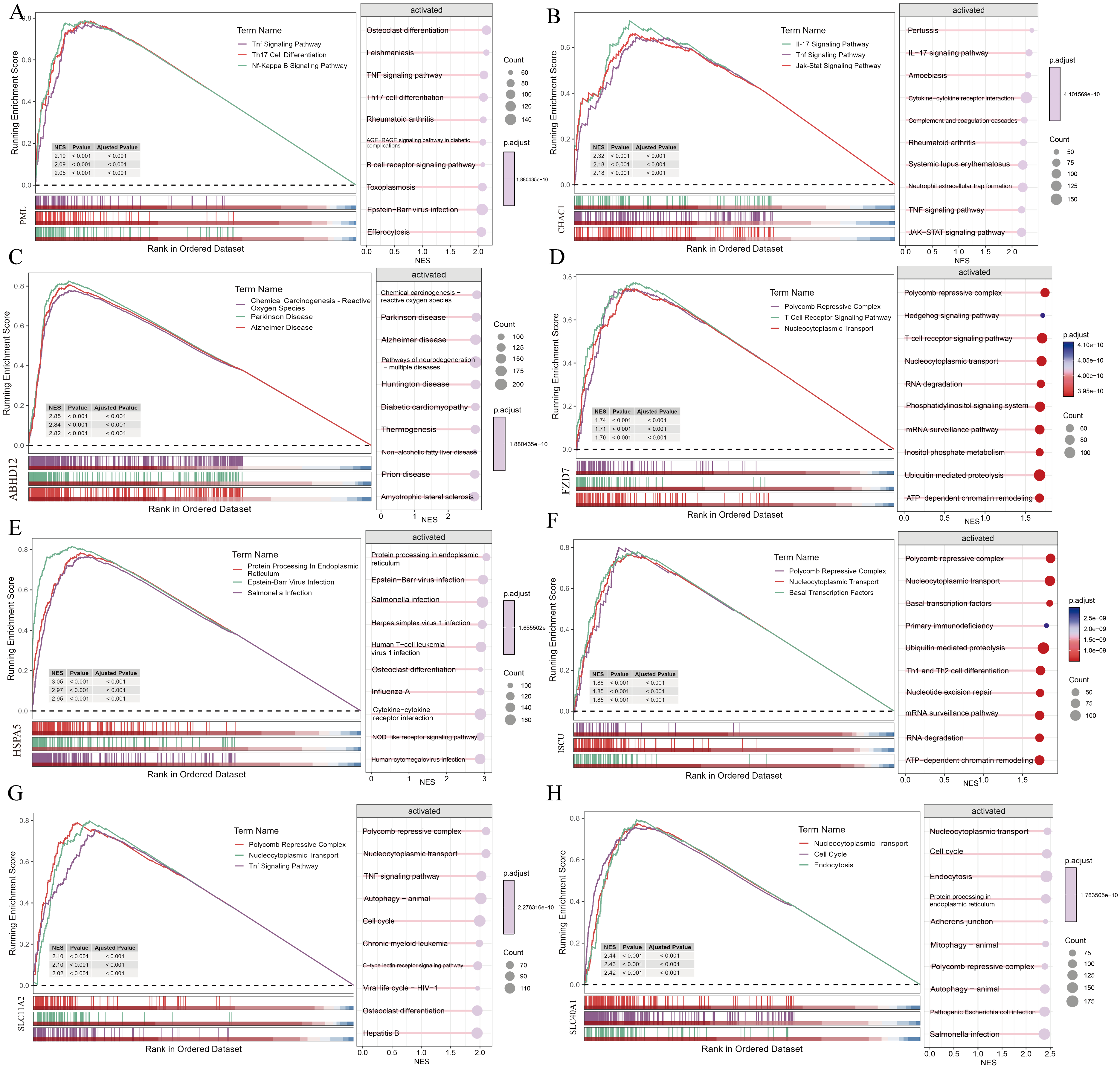

Supplement: Supplementary Figure 2 — (A–H) Pathways enriched after ranking the correlated genes of the 8 genes in GSE107794. [file Image2.tif]

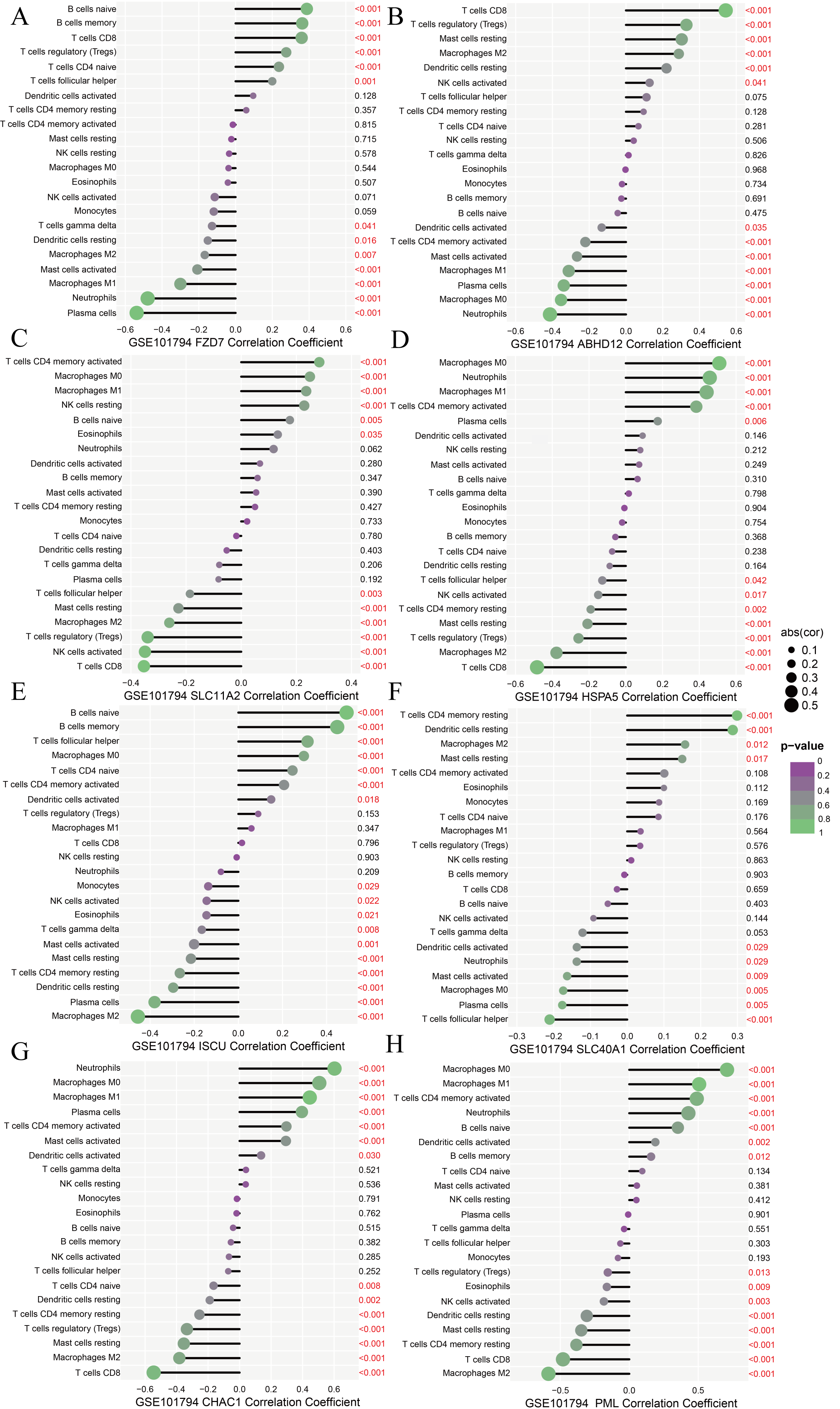

Supplement: Supplementary Figure 3 — (A–H) The abundance of infiltrating immune cells in GSE101794 using Spearman correlation analysis. The size of the point is proportional to the correlation strength, with the horizontal axis indicating positive or negative correlations, in the figure, groups with p < 0.05 are highlighted in red to indicate statistically significant differences. [file Image3.tif]

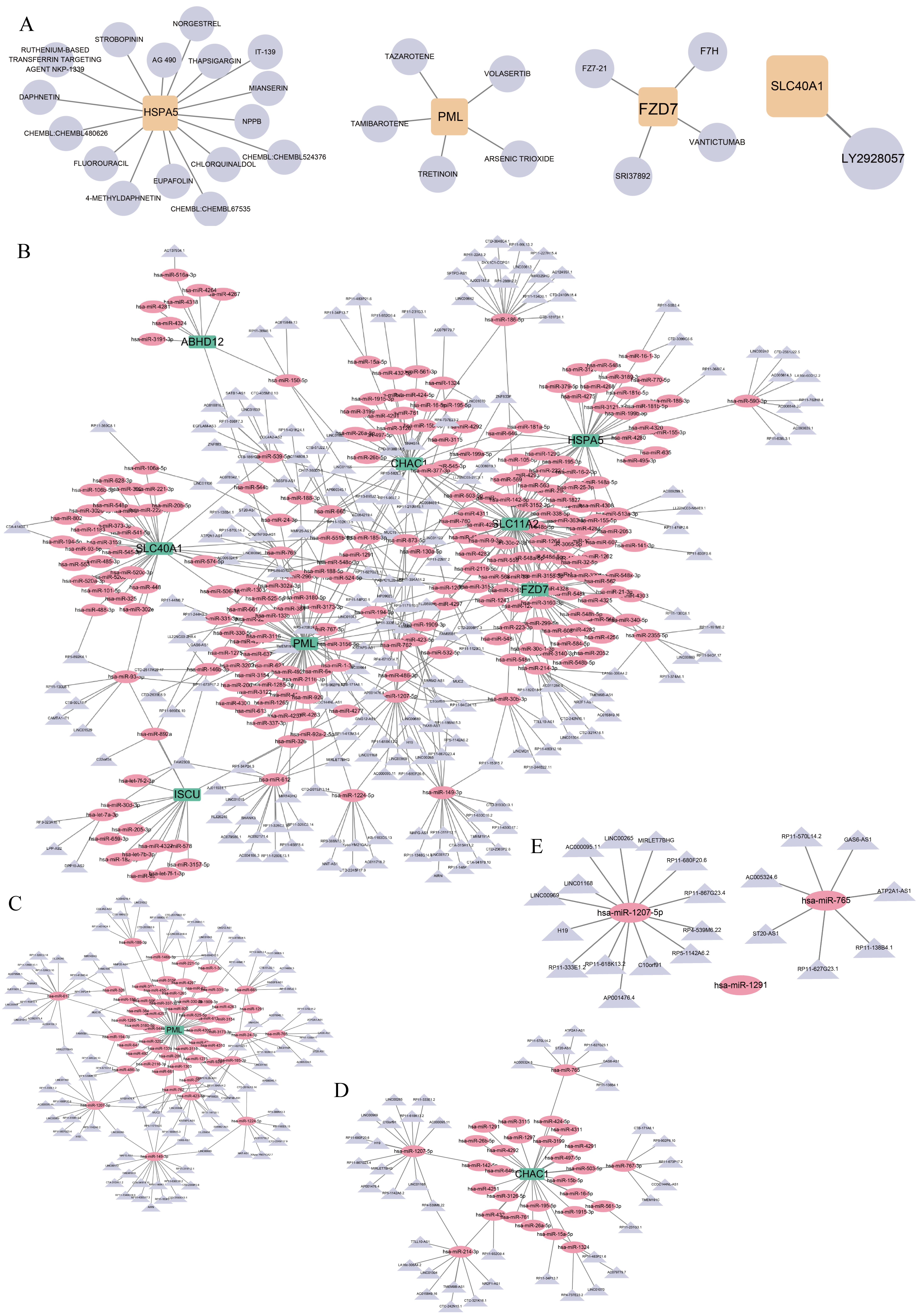

Supplement: Supplementary Figure 4 — (A) Prediction of targeted drugs for marker genes. (B) The ceRNA network, constructed based on the marker genes, comprises 445 nodes, interconnected by 549 edges. (C, D) The construction of a ceRNA network based on PML and CHAC1. (E) The overlapping nodes in the ceRNA networks of PML and CHAC1. [file Image4.tif]

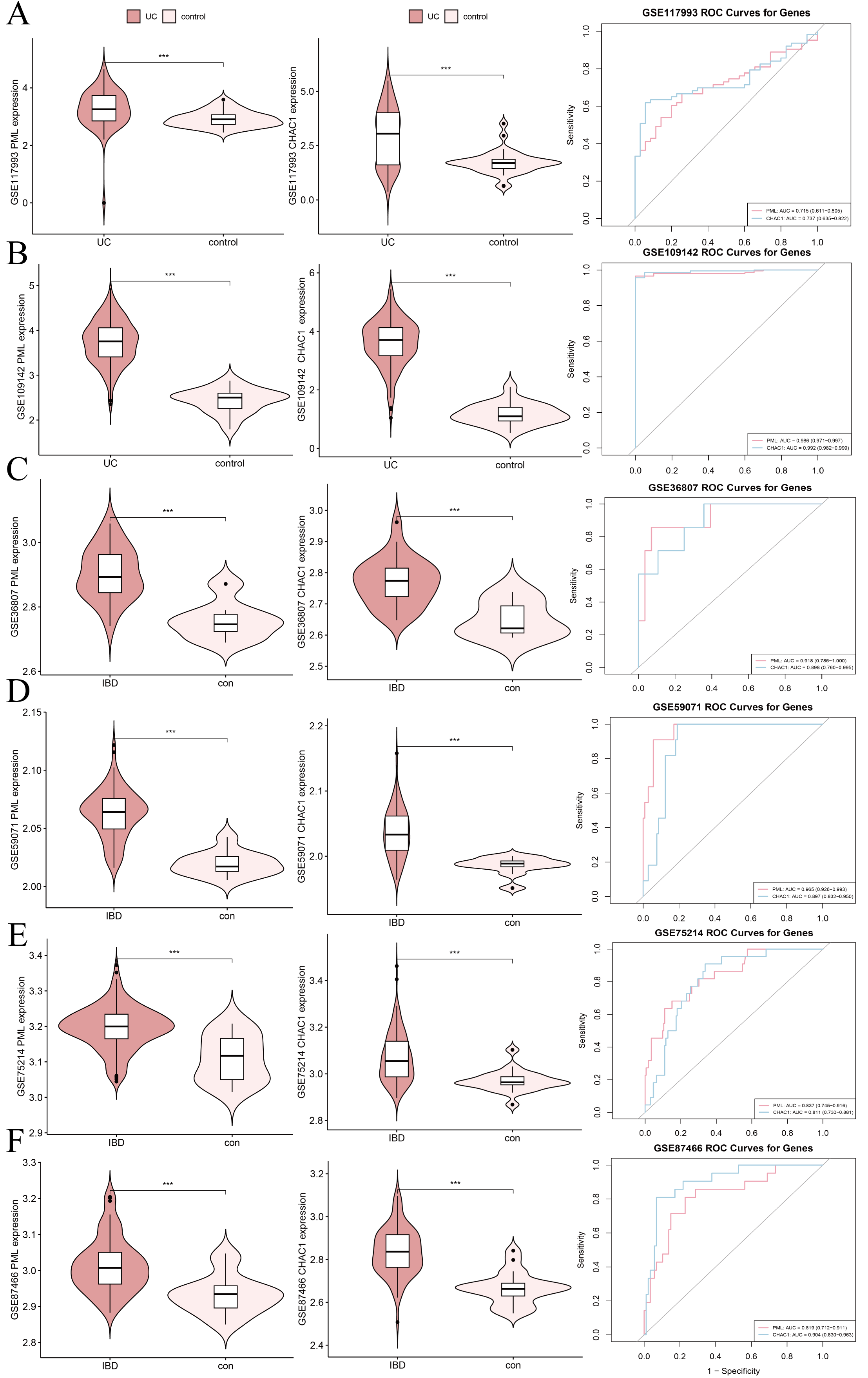

Supplement: Supplementary Figure 5 — Expression and diagnostic evaluation in pediatric UC and adult IBD. (A, B) Gene expression and ROC curves in pediatric UC datasets (***p < 0.001). (C–F) Expression and ROC analysis in adult IBD datasets (***p < 0.001). [file Image5.tif]
