# Supplementary material for: Identification of ferroptosis-genes associated with pediatric inflammatory bowel disease bioinformatics and machine learning approaches
Source: Front Immunol. 2025 Nov 12;16:1619944. doi: 10.3389/fimmu.2025.1619944 (PMC12647037; doi:10.3389/fimmu.2025.1619944)
Supplement: Supplementary file 7 [file Table2.docx]

| **Characteristic** | **Disease** | | | **p-value** |
| --- | --- | --- | --- | --- |
|  | **Overall  N = 20** | **Crohn's disease  N = 10** | **Control  N = 10** |  |
| **Age, n (%)** |  |  |  | 0.15^1^ |
| 13 | 1 (5.0%) | 0 (0.0%) | 1 (10.0%) |  |
| 14 | 6 (30.0%) | 2 (20.0%) | 4 (40.0%) |  |
| 15 | 6 (30.0%) | 2 (20.0%) | 4 (40.0%) |  |
| 16 | 4 (20.0%) | 4 (40.0%) | 0 (0.0%) |  |
| 17 | 3 (15.0%) | 2 (20.0%) | 1 (10.0%) |  |
| **Gender, n (%)** |  |  |  | >0.99^2^ |
| male | 10 (50.0%) | 5 (50.0%) | 5 (50.0%) |  |
| female | 10 (50.0%) | 5 (50.0%) | 5 (50.0%) |  |
| **Region, n (%)** |  |  |  | >0.99^1^ |
| Colon/Ileal | 6 (60.0%) | 6 (60.0%) | 0 (NA%) |  |
| Colon | 3 (30.0%) | 3 (30.0%) | 0 (NA%) |  |
| Ileal | 1 (10.0%) | 1 (10.0%) | 0 (NA%) |  |
| **Active, n (%)** |  |  |  | >0.99^1^ |
| yes | 9 (90.0%) | 9 (90.0%) | 0 (NA%) |  |
| no | 1 (10.0%) | 1 (10.0%) | 0 (NA%) |  |
| **Use of Biologic Agents, n (%)** |  |  |  | >0.99^1^ |
| yes | 9 (90.0%) | 9 (90.0%) | 0 (NA%) |  |
| no | 1 (10.0%) | 1 (10.0%) | 0 (NA%) |  |
| ^1^Fisher's exact test | | | | |
| ^2^Pearson's Chi-squared test | | | | |
